# An engineered HIV-1 Gag-based VLP displaying high antigen density induces strong antibody-dependent functional immune responses

**DOI:** 10.1038/s41541-023-00648-4

**Published:** 2023-04-06

**Authors:** Ferran Tarrés-Freixas, Carmen Aguilar-Gurrieri, María Luisa Rodríguez de la Concepción, Victor Urrea, Benjamin Trinité, Raquel Ortiz, Edwards Pradenas, Pau Blanco, Sílvia Marfil, Luis Manuel Molinos-Albert, Ana Barajas, Anna Pons-Grífols, Carlos Ávila-Nieto, Ismael Varela, Laura Cervera, Sònia Gutiérrez-Granados, María Mercedes Segura, Francesc Gòdia, Bonaventura Clotet, Jorge Carrillo, Julià Blanco

**Affiliations:** 1 IrsiCaixa AIDS Research Institute, Can Ruti Campus, 08916 Badalona, Catalonia Spain; 2 Comparative Medicine and Bioimage Centre of Catalonia (CMCiB), Germans Trias i Pujol Research Institute (IGTP), Can Ruti Campus, 08916 Badalona, Catalonia Spain; 3 Grup d’Enginyeria Cel•lular i Bioprocessos, Department of Chemical, Biological and Environmental Engineering, Escola d’Enginyeria, Universitat Autònoma de Barcelona, Campus de Bellaterra, 08913 Cerdanyola del Vallès, Catalonia Spain; 4 University of Vic–Central University of Catalonia (UVic-UCC), 08500 Vic Barcelona, Spain; 5 Germans Trias i Pujol Research Institute (IGTP), Can Ruti Campus, Badalona, 08916 Barcelona, Spain; 6 CIBER Enfermedades Infecciosas (CIBERINFEC), Instituto de Salud Carlos III, Madrid, Spain; 7 Present Address: ISGlobal, Hospital Clínic-Universitat de Barcelona, Barcelona, Spain

**Keywords:** DNA vaccines, Protein vaccines, Retrovirus, Antibodies, HIV infections

## Abstract

Antigen display on the surface of Virus-Like Particles (VLPs) improves immunogenicity compared to soluble proteins. We hypothesised that immune responses can be further improved by increasing the antigen density on the surface of VLPs. In this work, we report an HIV-1 Gag-based VLP platform engineered to maximise the presence of antigen on the VLP surface. An HIV-1 gp41-derived protein (Min), including the C-terminal part of gp41 and the transmembrane domain, was fused to HIV-1 Gag. This resulted in high-density MinGag-VLPs. These VLPs demonstrated to be highly immunogenic in animal models using either a homologous (VLP) or heterologous (DNA/VLP) vaccination regimen, with the latter yielding 10-fold higher anti-Gag and anti-Min antibody titres. Despite these strong humoral responses, immunisation with MinGag-VLPs did not induce neutralising antibodies. Nevertheless, antibodies were predominantly of an IgG2b/IgG2c profile and could efficiently bind CD16-2. Furthermore, we demonstrated that MinGag-VLP vaccination could mediate a functional effect and halt the progression of a Min-expressing tumour cell line in an in vivo mouse model.

## Introduction

Human Immunodeficiency Virus-1 (HIV-1) has developed several strategies to impair the development of protective immune responses. Among them, the low incorporation of envelope glycoproteins (Env) on the viral surface may result in reduced antibody avidity, which may hamper the development of potent neutralising Env-specific humoral immune responses^1,2^. The delivery of antigen at high-density on multivalent platforms is considered an important mean to induce potent B-cell responses both in natural infection or during vaccination^3–6^. Therefore, these types of strategies are progressively reaching the human vaccine field, with one recent example being the Novavax nanoparticle-based subunit vaccine against SARS-CoV-2 (NVX-CoV2373)^7^. Other strategies currently in development are based on synthetic nanoparticles, such as liposomes and Virus-like Particles (VLPs), or DNA/RNA delivery systems that are able to present a high number of membrane-bound antigens to naive B cells, improving their priming and supporting antibody maturation in germinal centres^8–17^. In this sense, HIV-1 Gag-based enveloped VLPs are a promising vaccine platform^17,18^.

Enveloped Gag-VLPs are non-infectious and non-replicative viral particles. Gag-VLPs are assembled at the cell membrane by oligomerisation of the HIV-1 p55Gag polyprotein releasing to the extracellular space particles that mimic virion structural features^18–20^. VLPs are currently being tested as HIV-1 vaccine candidates in preclinical animal models (mice, macaques and rabbits) and different formulations are evaluated: nucleic acids^21–23^, purified VLPs^24–27^ or heterologous strategies^28–30^. Although these studies have demonstrated that retroviral Gag-based VLPs are able to induce potent immune responses^31^, a limitation remains since HIV-1 Env is poorly incorporated on viral particles and VLPs^32^.

Strategies to increase antigen density on the surface of VLPs include the incorporation of multimerization tags^33^, the modification of the Env cytoplasmic tail^34^ or its substitution by those from other viral proteins^23^. In this work, we describe a high-density antigen-displaying HIV-1 Gag-based VLP platform generated by the fusion of an extracellular antigen to HIV-1 Gag via a transmembrane domain. A small HIV-1 gp41-derived antigen containing a fragment of the HR2 domain, the membrane proximal external region (MPER) and the gp41 transmembrane domain was selected as model antigen^35^. This antigen improves the exposure of the MPER^36^, which is one of the most conserved HIV-1 Env regions. In addition, anti-MPER neutralising antibodies (NAbs) are among the antibodies with the broadest neutralising activity (i.e., 10E8) described so far. Therefore, the MPER is an attractive target for HIV-1 vaccine development^35^. Theoretically, in our fusion-protein VLPs, the number of antigens displayed would be stoichiometrically equivalent to Gag (2500 Gag proteins/VLP)^19^ and far superior to the expected number of Env glycoproteins on the surface of HIV-1 virions (4–20 Env/virion^1,2^). Our VLPs induced a non-neutralising but potent and functional humoral immune response that could mediate a protective effect when used as a vaccine platform.

## Results

### MinGag-VLPs display similar morphology and composition as Gag-VLPs

**Fig. 1:**
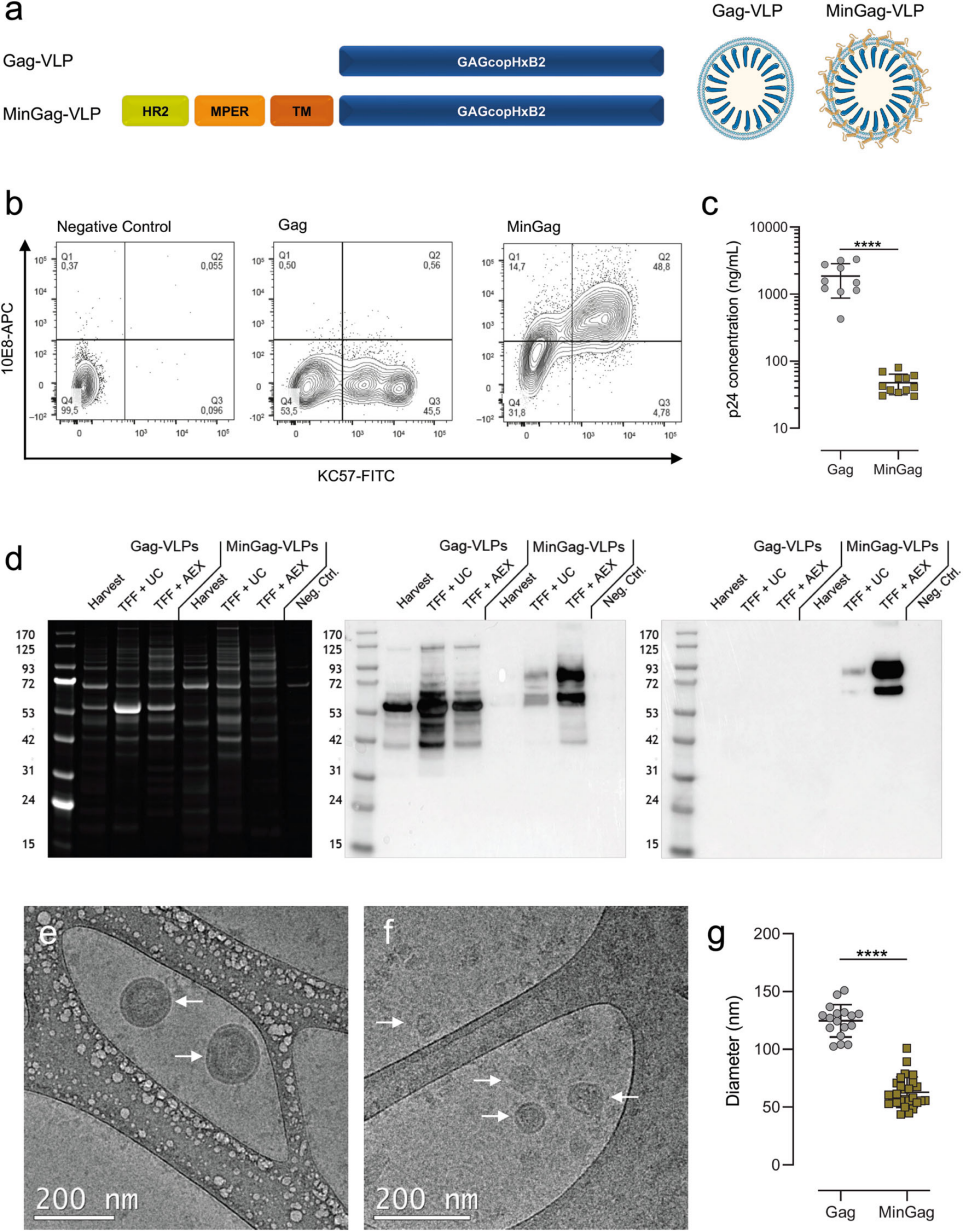
Characterisation of high-density HIV-1 Gag-based VLP production in Expi293F cells. **a** Schematic representation of the constructs and hypothetical structure of Gag-VLPs and high-density MinGag-VLPs. Min is a gp41-derived protein that contains a fragment of the HR2, MPER and TM domains. **b** FACS dot plots for the staining of non-transfected (left), *gag*-transfected (middle) and *mingag*-transfected (right) Expi293F cells stained with the anti-p24 KC57-FITC-conjugated mAb (X-axis) and the anti-MPER 10E8 antibody (*Y* axis). **c** Quantification of p24 by ELISA on harvested supernatants of *gag*-transfected and *mingag*-transfected Expi293F cells. Data represented as mean ± SD. Significant differences on production were found using a Mann–Whitney *U* test (****p < 0.0001). **d** Characterisation of HIV-1 Gag-VLPs and MinGag-VLPs from harvested supernatants and after purification with either sucrose-cushion ultracentrifugation (TFF + UC) or anion exchange chromatography (TFF + AEX) by Coomassie blue staining (left), anti-p24 WB (middle) and anti-2F5 WB (right). Both western blots correspond to the same gel. Uncropped images are shown at Supplementary Fig. 2a. **e** Cryo-EM of purified Gag-VLPs. Scale bar = 200 nm. **f** Cryo-EM of purified MinGag-VLPs. Scale bar = 200 nm. **g** Diameter comparison of Gag-VLP (*n* = 17) and MinGag-VLP (*n* = 27). Significant differences on size were found using a Mann–Whitney *U* test (****p < 0.0001). Data represented as mean ± SD.

Plasmids encoding HIV-1 Gag or the fusion-protein MinGag were transiently transfected into Expi293F cells to produce Gag-VLPs and MinGag-VLPs, respectively (Fig. 1a). Min antigen was efficiently detected by the anti-MPER 10E8 antibody on the surface of cells (Fig. 1b), while intracellular co-staining with an anti-p24 Gag antibody (KC57-FITC) confirmed the co-expression of Gag. Cells transfected with *gag*, and mock cells were used as controls (Fig. 1b). Gates are shown in Supplementary Fig. 1

Gag-VLPs and MinGag-VLPs were successfully recovered from the supernatant of transfected cells, but p24 Gag concentration was 10-fold lower for the latter (Fig. 1c), suggesting a lower MinGag-VLP production. SDS-PAGE and WB protein analyses showed that Gag-VLPs were only detected using anti-p24 antibodies (Fig. 1d and Supplementary Fig. 2), whereas MinGag fusion-protein was detected by both anti-p24 and anti-MPER antibodies. MinGag protein had the expected apparent molecular weight of 72 kDa. However, some degradation products were detected by both anti-p24 and anti-MPER antibodies, suggesting partial fragmentation or processing of the fusion-protein (Fig. 1d). Despite the lower concentration of MinGag-VLPs compared to Gag-VLPs in the supernatant, both were effectively purified by a two-step protocol involving either TFF + UC or TFF + AEX (see methods). Considering both procedures, TFF + UC yielded superior recoveries, but for MinGag-VLPs, TFF + AEX resulted in a 10-fold increase of sample purity as assessed by the ratio of p24 protein over the total protein content of these preparations (Fig. 1d, and Supplementary Table 1). As expected, Cryo-EM analysis of purified Gag- and MinGag-VLPs revealed a morphology of round-shaped particles with a lipid membrane containing a p55Gag electrodense core (Fig. 1e, f). However, compared to Gag-VLPs, the diameter of MinGag-VLPs was significantly reduced (Fig. 1g, 124.7 ± 13.6 nm vs. 62.9 ± 13.0 nm; *p* < 0.0001).

### Impact of transmembrane and linker domains on antigen exposure and density

**Fig. 2:**
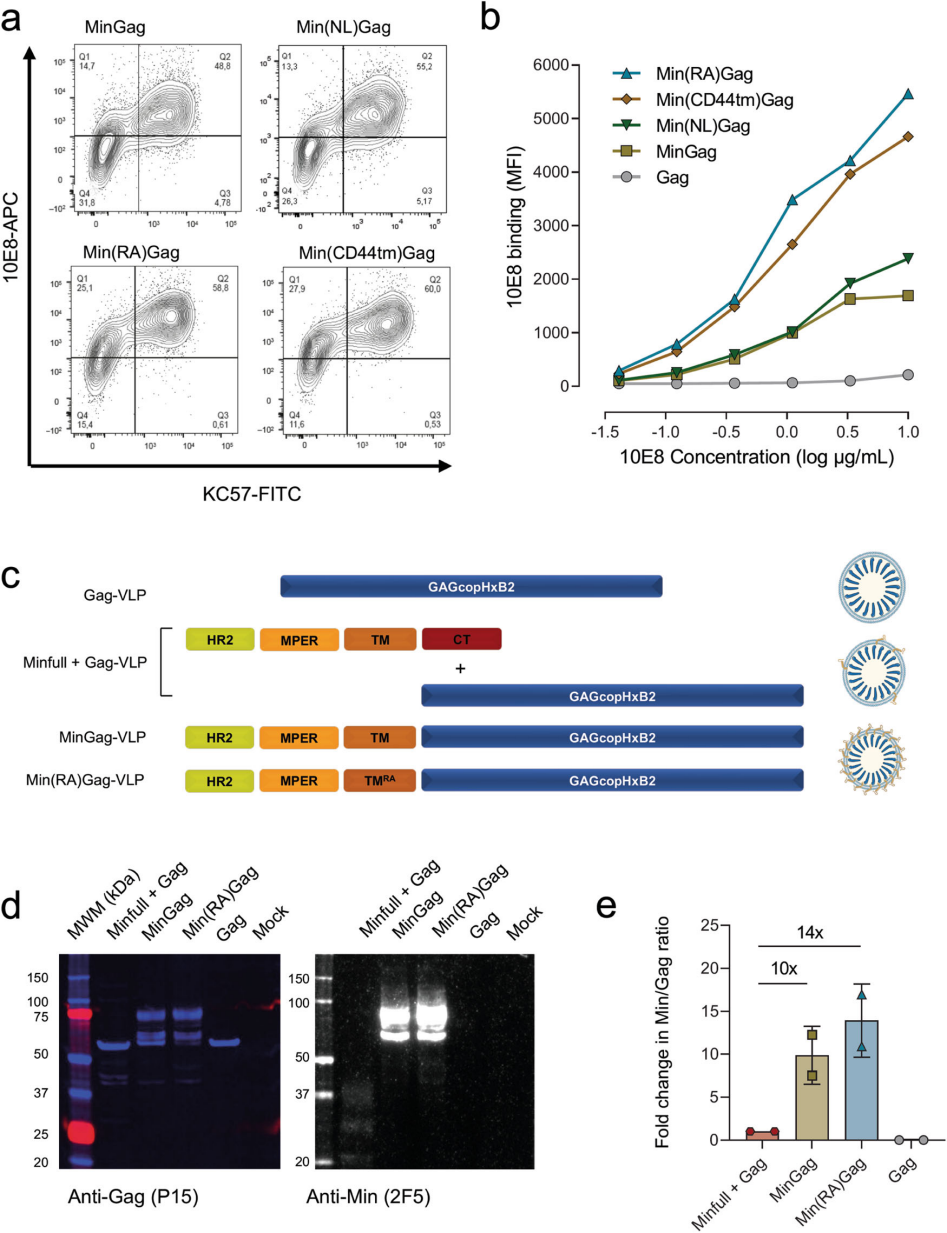
Optimisation of Min antigenicity on the surface of VLP-producing Expi293F cells. **a** Dot plots of Expi293F cells transfected with *mingag* constructs bearing different transmembrane or linker variants: original (MinGag; same as shown in Fig. 1b) no linker (Min(NL)Gag), gp41 R696A mutation (Min(RA)Gag), CD44tm (Min(CD44)Gag). Cells were extracellularly stained with an anti-MPER antibody (10E8) and intracellularly stained with an anti-p24 antibody (KC57-FITC). **b** Dose-response curves of 10E8-binding to Min on the surface of Expi293F cells transfected with transmembrane and linker *mingag* variants. **c** Schematic representation of the strategies used to produce Gag-VLPs, low-density Minfull + Gag-VLPs or high-density MinGag- and Min(RA)Gag-VLPs. **d** Western Blot analysis of the same gel loaded with VLPs purified from supernatant of Expi293 cells transfected with Minfull + Gag, MinGag or Min(RA)Gag. Left panel: anti-p15 antibody. Right panel: 2F5 antibody (anti-MPER). Uncropped images are displayed at Supplementary Fig. 2b. **e** Quantification of Min/Gag ratios from the Western Blot as a measure of Min incorporation into VLPs. Data represented as mean ± SD, and values indicate fold change.

To optimise antigen exposure on the surface of VLPs, different MinGag transmembrane and linker modifications were tested. Such variations included the substitution of the transmembrane domain of gp41 for the transmembrane domain of the human CD44 protein, which mostly localises in cholesterol-rich microdomains^37^. Furthermore, we also evaluated the effect of substituting the arginine at position 696 in the gp41 wildtype transmembrane domain by an alanine (R696A). This arginine has been described to mediate conformational changes during HIV-1 fusion with the host cell^38^, and hence it could impact the antigen display. Besides the modifications in the transmembrane domain, the original GGGS flexible linker between Min and Gag was also removed (no linker, NL) to evaluate its impact on VLP antigenicity. Of note, binding of 10E8 on the surface of VLP-producing cells demonstrated that the construct with the R696A substitution (Min(RA)Gag) and Min(CD44tm)Gag led to a better antigenic exposure. In contrast, the removal of the linker had no effect on antigenicity (Fig. 2a). Accordingly, MinGag and Min(RA)Gag constructs were selected since they only displayed HIV-1 derived sequences and provided two different levels of antigenicity (Fig. 2b).

In addition, we also evaluated the antigenic density of final VLP preparations. For comparative purposes, we produced standard VLPs by co-transfection of Expi293 cells with plasmids encoding Gag protein and a Min protein containing the intracellular domain of gp41^36^. This experimental setting mimics natural antigen incorporation to VLPs. A nude Gag-VLP was also produced (Fig. 2c, and Supplementary Fig. 2). Western blot analysis showed a lower content of Min antigen in standard VLPs compared to MinGag and Min(RA)Gag-VLPs (Fig. 2d). Quantitative analysis confirmed that the Min density in MinGag and Min(RA)Gag-VLPs was 10- and 14-fold higher, respectively, than for standard VLPs (Fig. 2e).

### Induction of a Th1-like humoral response by MinGag-VLPs

Immunisation strategies combining heterologous formulations of the same antigen have proven to elicit superior immune responses^39^ and have reached massive testing in humans during COVID-19 pandemics^40^. Therefore, we assessed the immunogenicity of MinGag-VLPs and Min(RA)Gag-VLPs in C57BL/6JOlaHsd mice following two different strategies: a homologous regimen using four doses of purified VLPs (VVVV) or a combined/heterologous regimen priming with two doses of DNA and boosting with two doses of purified VLPs (DDVV). Gag-VLPs and PBS were included as controls.

**Fig. 3:**
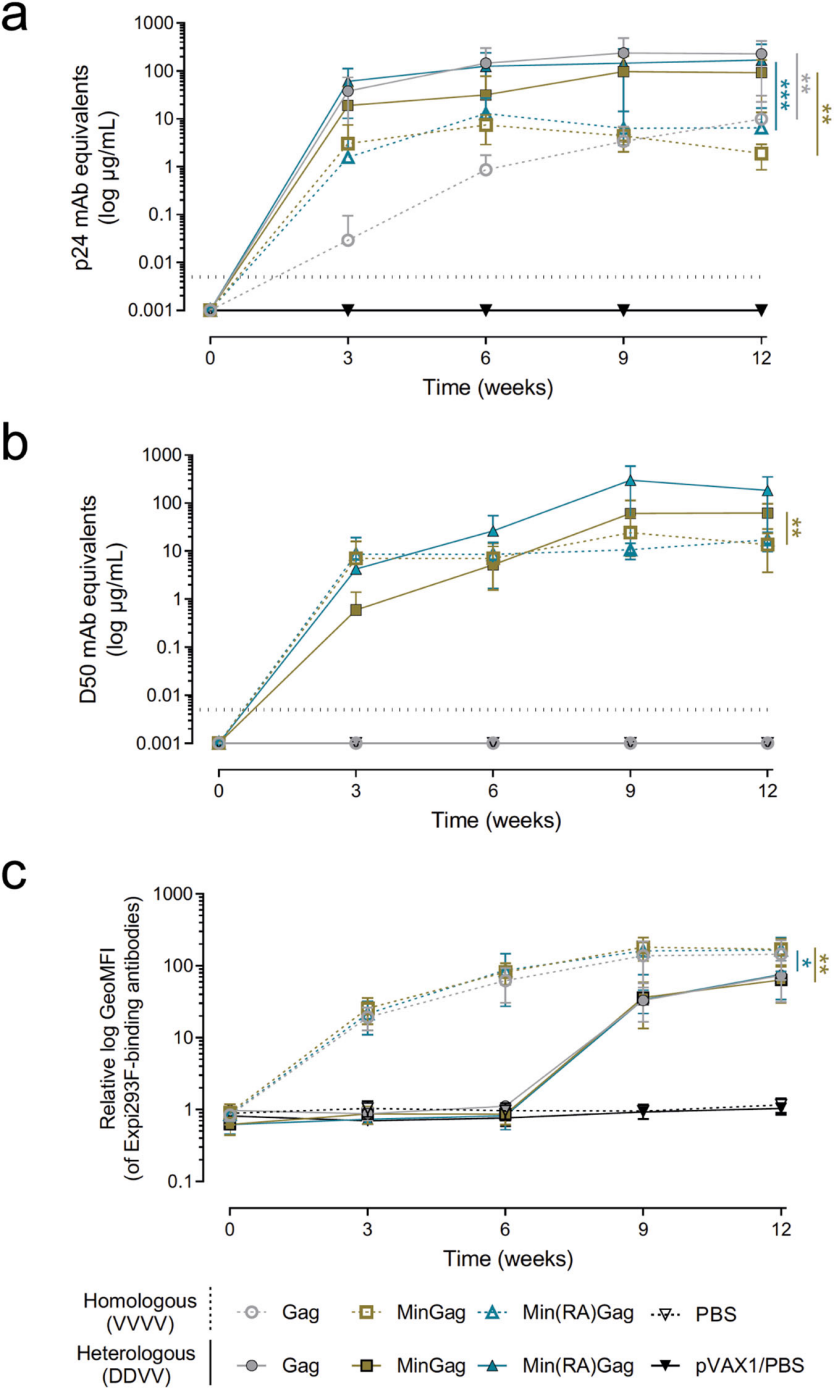
Humoral responses induced by homologous and heterologous immunisation regimens. C57BL/6JOlaHsd mice were immunised at weeks 0, 3, 6, and 9 with a homologous (VVVV; dashed line) or heterologous (DDVV; solid line) regimens consisting on Gag-VLPs (grey rounds; *n* = 10), MinGag-VLPs (gold squares; *n* = 10) or Min(RA)Gag-VLPs (blue triangles; *n* = 10) and a pVAX1/PBS control or PBS control (black triangles; *n* = 4). **a** Concentration of anti-Gag IgG in mouse sera determined by ELISA. **b** Concentration of anti-Min IgG in mouse sera determined by ELISA. **c** Concentration of IgGs against Expi293F host cell proteins analysed by FACS. Data are presented as mean ± 95%CI for the ELISA results and geo-MFI ± SD for the FACS results. Statistical differences at week 12 were found using a Kruskal–Wallis test with Dunn’s correction (*p < 0.05; **p < 0.01; ***p < 0.001).

Overall, both strategies induced both anti-Gag and anti-Min IgG antibody responses (Fig. 3a, b). Vaccination with purified VLPs did not induce potent antibody responses against Gag (Fig. 3a). Interestingly, DDVV vaccination induced a 10-fold higher IgG concentration in serum at endpoint compared to VVVV for both the anti-Gag and anti-Min responses (Fig. 3a, b). Anti-Gag responses in DDVV and VVVV (Fig. 3a), as well as anti-Min responses in VVVV (Fig. 3b), reached the maximal IgGs concentration in serum 3 weeks after the first immunisation. In comparison, concentration of anti-Min IgGs in animals vaccinated with MinGag-VLPs and Min(RA)Gag-VLPs in the DDVV regimen increased after each DNA shot and after the first VLP immunisation. However, significant differences compared to the VVVV regimen were only observed for the MinGag-VLP group (Fig. 3b) due to a high dispersion in the Min(RA)Gag-VLP group. Min(RA)Gag induced a slightly superior, although non-significant, anti-Min IgG concentration than MinGag in the DDVV regimen. While no significant differences were detected between females and males, a trend towards higher IgG concentrations was observed in the former (Supplementary Fig. 3a–d).

As VLPs were produced in a human cell line, human host proteins were expected to be incorporated into the VLPs. Indeed, mice immunised with purified VLPs elicited antibody responses against human proteins (Fig. 3c). In the VVVV regimen, IgG titres against human proteins progressively increased, reaching a plateau after the third dose. In contrast, in the DDVV regimen, no anti-Expi293F protein responses were detected after DNA immunisations, since VLPs were produced directly in vivo by murine cells (Fig. 3c), while similar kinetics of anti-Expi293F antibodies were observed after subsequent VLP immunisation.

Epitope mapping showed that anti-Min antibodies preferentially targeted the N-terminus region of Min, which contains a fragment of the HR2 domain (Supplementary Fig. 4a–c). However, in the DDVV regimen, anti-Min IgG antibodies also targeted the C-terminus of the HR2 domain (Supplementary Fig. 4b).

**Fig. 4:**
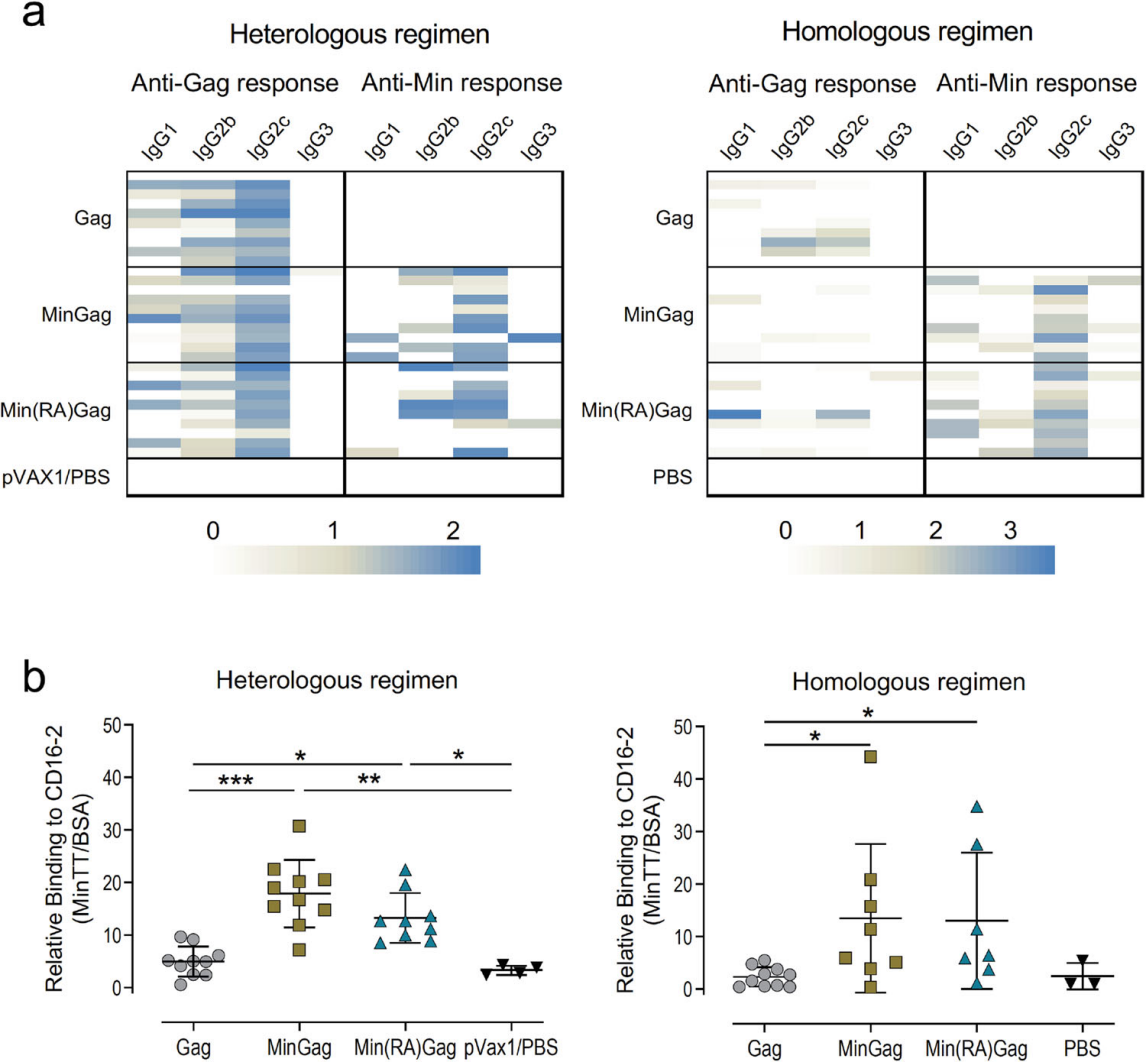
Functional properties of VLP-induced humoral responses. Serum samples at week 12 from C57BL/6JOlaHsd mice vaccinated with heterologous (left panels) or homologous (right panels) regimens consisting of Gag-VLPs (grey; *n* = 10), MinGag-VLPs (gold; *n* = 10), Min(RA)Gag-VLPs (blue; *n* = 10) and pVAX1/PBS or PBS controls (black, *n* = 4). **a** Heatmaps of the predominant murine IgG subclasses (IgG1, IgG2b, IgG2c, or IgG3) for both anti-Gag and anti-Min humoral responses in both vaccination regimens. Each line represents one animal. **b** Binding to mFcγRIV/CD16-2 by Min-specific antibodies in serum from VLP-immunised mice collected at week 12. Data represented as mean ± SD of relative binding, this being the absorbance values of CD16-2 binding of each serum sample against Gp41-MinTT referred to its absorbance against BSA. Significant differences were found using a Kruskal–Wallis test with Dunn’s comparison (*p < 0.05; **p < 0.01; ***p < 0.001).

Detection of the IgG subclass profile demonstrated that VLP-induced Gag-specific antibodies were heterogeneous (IgG1, IgG2b and IgG2c), while Min-specific antibodies were mainly IgG2c (Fig. 4a), regardless of the vaccination regimen. IgG subclasses are relevant for Fc-mediated functions, since murine IgG2c and, to a lesser extent, IgG2b can bind to the NK cell activating receptor mFcγRIV (CD16-2)^41^. Consistently, anti-Min antibodies bound to CD16-2 receptor (Fig. 4b), indicating that they could mediate Fc-effector functions such as antibody-dependent cell cytotoxicity (ADCC)^42,43^.

Neutralisation capacity of VLP-induced antibodies was tested in vitro against 3 subtype B HIV-1 pseudoviruses (NL4.3, AC10.0.29 and TRO.11), an HIV-2/HIV-1 MPER chimera (7312 A/C1), and an HIV-2 control (7312 A) showing no significant neutralising activity (Supplementary Fig. 5a, b).

T-cell responses against Min and Gag proteins were evaluated by IFN-γ ELISpot using cryopreserved murine splenocytes. As anticipated, T-cell responses were mostly directed against Gag, albeit no significant differences were found between VLP constructs (Supplementary Fig. 6a, b). Moreover, no T-cell responses were detected against the MPER^44,45^, whereas weak responses were elicited against Min in MinGag-VLP-immunised animals in the heterologous regimen (Supplementary Fig. 6a, b).

### In vivo functional protection assay

**Fig. 5:**
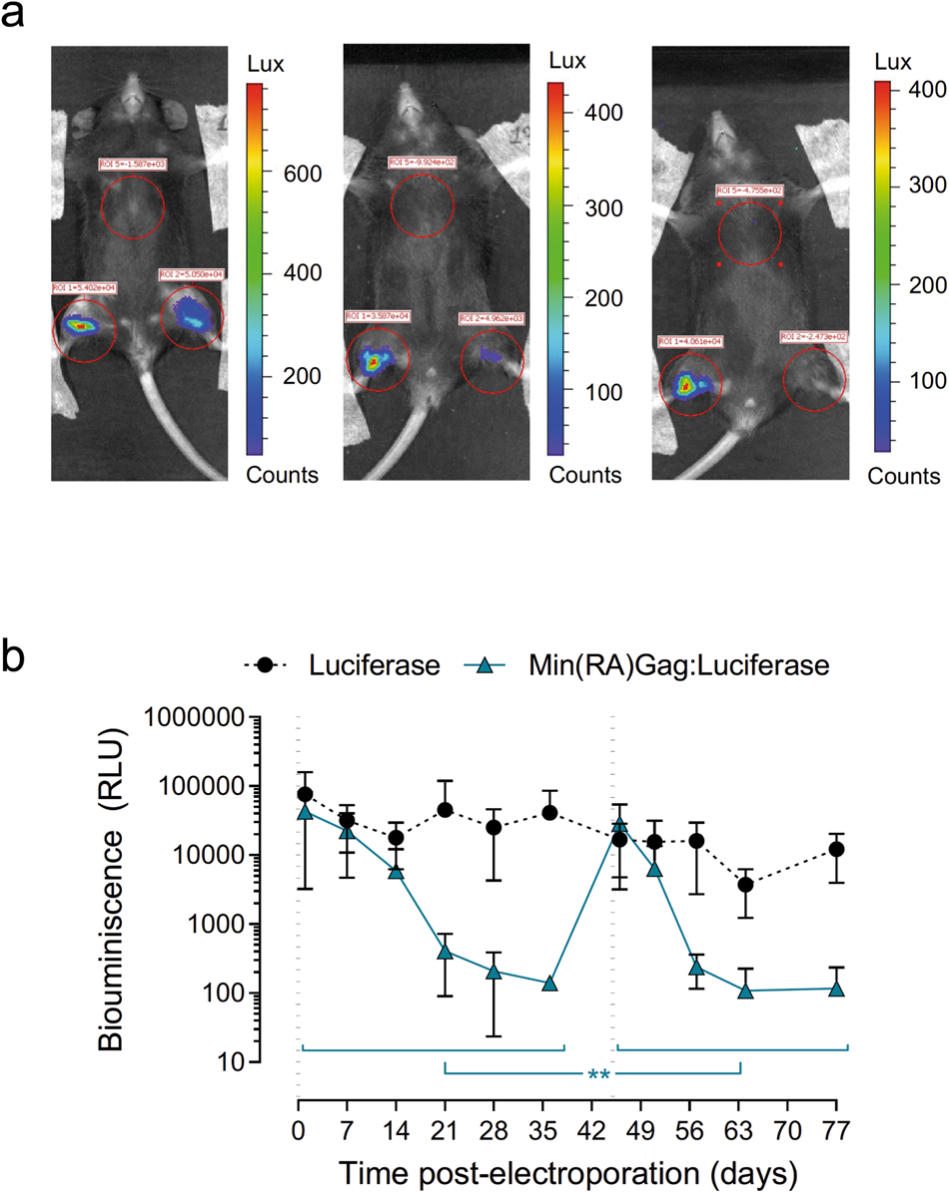
In vivo bioluminescence kinetics of DNA co-electroporation in mice. C57BL/6JOlaHsd mice (*n* = 5) were electroporated with 20 µg of DNA of both a 1:1 mix of pVAX1:pVAX1-*Fluc* at the right limb (black rounds) at day 0 and a 1:1 mix of pVAX1-*Fluc*:pVAX1-*min(RA)gag* at the left limb (blue triangles) at day 0 and 45. **a** Representative bioluminescence images of co-electroporated mice at 48 h (left), 7 days (middle) and 14 days (right) post-electroporation. **b** Bioluminescence follow-up of electroporated mice. Data represented as mean ± SD. Significant differences between the first and the second electroporation (days 0–35 and 46–77, respectively) at the left limb (pVAX1-*Fluc*:pVAX1-*min(RA)gag*) were assessed comparing nested mixed effect models and considering time as a categorical factor (**p = 0.0012; likelihood ratio test).

To evaluate whether VLPs could induce a functional immune response in vivo, C57BL/6JOlaHsd mice were electroporated with plasmids encoding Luciferase (pVAX1-*FLuc*:pVAX1 at 1:1 ratio in the right hindlimb) or Luciferase+Min(RA)Gag (pVAX1-*FLuc*:pVAX1-*min(RA)gag* at a 1:1 ratio in the left hindlimb) (Fig. 5a). A specific effector immune response against Min was expected to induce a faster clearance of the luciferase expression in Min(RA)Gag-immunised animals compared to control animals as a consequence of the elimination of Min-expressing cells. Bioluminescence analysis demonstrated, on the one hand, a stable production of luciferase over time (up to 77 days after electroporation) in control animals, indicating that anti-luciferase immunogenicity was minimal and/or did not affect its expression. On the other hand, Luciferase+Min(RA)Gag co-electroporated muscles showed a decrease in luminescence that was evident 3 weeks after electroporation (Fig. 5b). Interestingly, the decrease in luciferase expression was faster after a second co-electroporation with Luciferase+Min(RA)Gag (*p* = 0.0012). Taken together, these data suggest that pVAX1-*min(RA)gag* could induce an effector immune response that cleared VLP-producing cells.

**Fig. 6:**
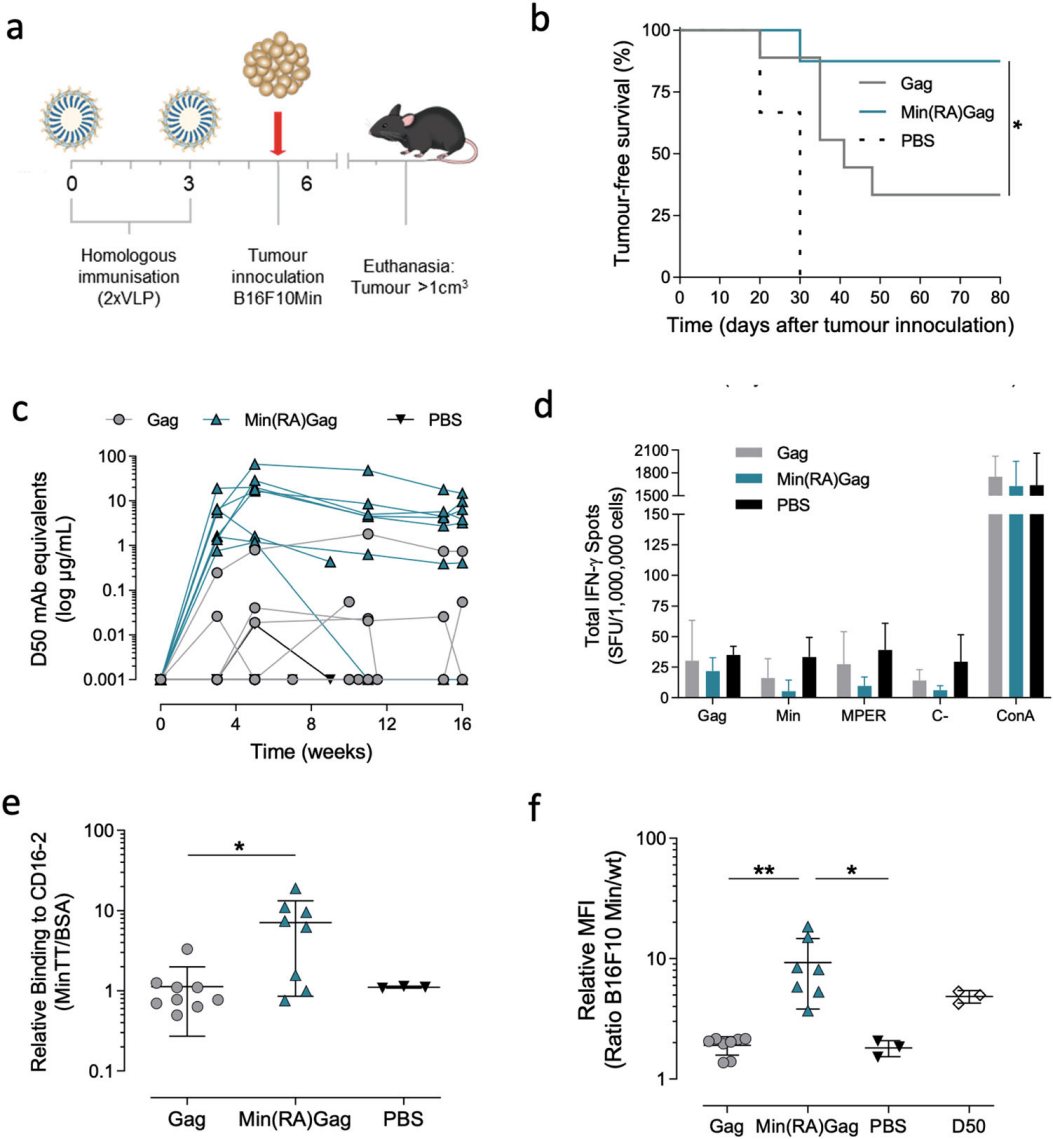
In vivo protection assay to identify vaccine-induced protection. **a** Schematic representation of the experimental model. C57BL/6JOlaHsd mice were immunised twice with Gag-VLPs (grey rounds; *n* = 9), Min(RA)Gag-VLPs (blue triangles; *n* = 8), or PBS controls (black inverted triangles; *n* = 3) at weeks 0 and 3. At week 5, animals were inoculated with a 100,000 B16F10Min cells and followed up for a period of 80 days after tumour inoculation. **b** Kaplan–Meier curves of tumour-free survival in VLP vaccinated and tumour-inoculated mice. Animals were euthanised for humane criteria when the tumour reached a size bigger than 1000 mm^3^. Significance was detected between Gag-VLP and Min(RA)Gag-VLP groups with a Mantel–Cox test (*p = 0.0414). **c** Anti-Min IgG concentration in the serum of VLP-immunised and tumour-inoculated mice determined by ELISA. **d** Cellular immune response against pools of Gag, Min, and MPER using splenocytes from vaccinated and tumour-inoculated mice and collected at endpoint. Data represented as mean ± SD. No significant differences were found in panels **c**–**e** between vaccinated groups using a Kruskal–Wallis test. **e** Relative binding to FcγRIV/CD16-2 of anti-Min antibodies referred to a BSA control. Serum was collected at week 5. Data represented as mean ± SD. Significant differences were found with a Kruskal–Wallis test with multiple comparison correction (*p ≤ 0.05). **f** Binding capacity to B16F10Min cells of antibodies induced by VLP-induced by VLP vaccination in serum from week 4. Data represented as mean ± SD of relative MFI of each serum sample binding to B16F10Min cells referred to their respective B16F10 control. Significant differences were found in using a Kruskal-Wallis test with Dunn’s comparison (*p < 0.05, **p < 0.01).

To functionally characterise anti-Min antibody responses, C57BL/6JOlaHsd mice were vaccinated with two doses of Gag-VLPs or Min(RA)Gag-VLPs. In this setting, a homologous VLP regimen was preferred over a heterologous DNA/VLP regimen to test a more conventional approach with a shorter vaccination schedule. Two weeks after the second immunisation, mice were inoculated with a B16F10 melanoma clone that stably expressed the Min protein on its surface (Supplementary Fig. 7a–c, Fig. 6a). The B16F10 cell line was chosen because of its poor immunogenicity^46^. After tumour inoculation, animals were monitored for up to 80 days. Unvaccinated mice started to show detectable tumour growth approximately 10 to 15 days after B16F10Min cell inoculation (Supplementary Fig. 8a). In comparison, the detection of tumour growth in animals vaccinated with either Gag-VLPs and Min(RA)Gag-VLPs was delayed (20–25 days post-tumour inoculation) (Supplementary Fig. 8a). Importantly, Min(RA)Gag-VLP vaccination significantly impacted tumour progression (Fig. 6b, *p* = 0.0414) compared to Gag-VLP-vaccinated animals, and tumour growth was detected only in one out of eight Min(RA)Gag-vaccinated mice, compared to six out of nine Gag-vaccinated animals that reached the humane endpoint.

Immune profiling of the IgG responses against Gag (Supplementary Fig. 8b), Min (Fig. 6c) and Expi293-derived proteins (Supplementary Fig. 8c) and the cellular responses (Fig. 6d) induced in Gag-VLP and Min(RA)Gag-VLP vaccinated, and tumour-inoculated animals matched with our previous vaccination results (Fig. 3). Concentration of anti-Min IgG was not significantly boosted after tumour inoculation (Fig. 6b). As anticipated, no significant neutralisation activity was detected in immunised and tumour-inoculated mice (Supplementary Fig. 8d), and anti-Min antibodies presented predominantly an IgG2c subclass (Supplementary Fig. 8e) that could bind to the CD16-2 receptor (Fig. 6e). Furthermore, anti-Min antibodies were able to bind B16F10Min cells (Fig. 6f). Interestingly, the Min(RA)Gag-vaccinated mouse that had to be euthanised due to uncontrolled tumour progression showed the lowest anti-Min antibody titre against B16F10Min cells and the lowest binding to CD16-2 at endpoint (Fig. 6e, f). Altogether, these results showed that Min(RA)Gag-VLP could induce an in vivo functional immune response mediating protection, possibly through antibody-dependent effector functions.

## Discussion

Strategies to potentiate immune responses against weak immunogens include antigen modifications^47^, intrastructural help^48^, and multivalent antigen display involving complex structures such as VLPs^8,49^. Enveloped HIV-1 Gag-based VLPs mimic the virus morphology and therefore may present immunogens in a more native fashion^9^. Multiple strategies to increase immunogen density on the VLPs’ surface have been developed^23^. Here, we present a strategy based on the fusion of an HIV-1 gp41-based immunogen to HIV-1 Gag via the gp41 transmembrane domain. By fusing the immunogen to Gag, the surface of the VLP was expected to be densely covered with it, since it would theoretically display as many antigens as Gag molecules (about 2500 molecules of Gag/VLP)^20^. This hypothesis was supported by the fact that MinGag and Min(RA)Gag-VLPs showed 10- to 14-fold higher Min/Gag ratios compared to VLPs obtained by co-transfection of Min and Gag in separate plasmids, confirming the positive impact on antigen density of our approach. Furthermore, FACS and quantitative WB analyses showed that the antigen was properly displayed on the surface of VLP-producing cells and that the MinGag fusion-protein was recognised by both anti-p24 and anti-gp41 antibodies. Besides demonstrating that MinGag fusion-protein was properly expressed in transfected cells, we also showed that MinGag-VLP morphology is similar to Gag-VLPs but with a reduced diameter and production yield. These differences could be a consequence of a premature association of Gag to the lipid membranes in VLP-producing cells. This premature membrane association of the MinGag nascent protein could promote intracellular accumulation that negatively impacts the release of VLPs. Thus, the concentration of MinGag-VLPs in the supernatant of VLP-producing cells was considerably lower than that of Gag-VLPs. Structural elements of the fusion proteins (transmembrane domain and linker) were evaluated in order to improve the immunogen exposure on the VLP membrane. Interestingly, the introduction of a single point mutation (R696A) into the gp41 transmembrane domain, which is thought to stabilise the transmembrane domain^38^, increased the antigen exposure on the surface of the VLP-producing cells, improving the MPER recognition by the 10E8 antibody.

Immunogenicity studies performed in C57BL/6JOlaHsd mice demonstrated that Min(RA)Gag-VLPs were immunogenic in vivo when administered as DNA vaccine or as purified VLPs. Comparison of a homologous VLP regimen (VVVV) and the heterologous DNA/VLP regimen (DDVV) showed that the combined regimen induced stronger anti-Gag and anti-Min humoral responses, in line with recent observations in humans for SARS-CoV-2 vaccination combining different vaccines^50,51^. Anti-Gag and anti-Min humoral responses in the VVVV regimen did not increase after each vaccination, but in the DDVV regimen these responses were properly boosted. The inferior performance of the VVVV regimen could be explained by the fact that, after the first dose, VLP administration mainly boosted humoral responses against human proteins derived from VLP-producing cells. In the DDVV regimen, these “anti-vector” responses were delayed, and anti-Min responses were boosted more efficiently. Furthermore, the fact that purified VLPs, both in the homologous regimen or as a booster in the heterologous regimen, induced lower anti-Gag responses compared to anti-Min responses further supports that MinGag-VLPs were properly formed in a particulate manner. Overall, the potent humoral response induced by MinGag-VLPs against Min was remarkable, especially considering that the C57BL/6 mouse model is biased towards Th1 immune responses^52^.

Besides the differences in antibody magnitude, the profiles of humoral responses were similar between the two regimens. Both targeted the N-terminus part of Min, which contains the HR2 peptide. This HR2 dominance could explain the absence of neutralising responses, and indeed, we could not induce antibodies against the MPER which is the primary target of gp41-specific broadly neutralising antibodies (bNAbs)^35^. Notwithstanding, anti-Min antibodies were mainly IgG2c, which is functionally equivalent to human IgG1, and efficiently bound to the murine CD16-2 receptor (mFcγRIV)^41,43^. Fc binding to CD16-2 in mice, as well as binding to CD16A in humans, activates NK cells and promotes ADCC. Therefore, our results suggest that VLP-induced anti-Min antibodies could mediate protective responses beyond neutralisation. In contrast, there was no predominant IgG subclass among anti-Gag antibodies in both immunisation regimens, suggesting that antigens displayed on the surface or within the VLP could induce qualitatively distinct antibody responses.

To investigate whether VLP-induced responses could mediate protection, we performed two in vivo experiments. First, mice were electroporated with a plasmid that coded for luciferase alone or combined with a plasmid that coded for Min(RA)Gag. Luciferase was detected for almost 3 months when the plasmid was electroporated alone. Conversely, when both plasmids were co-administered, luciferase signal progressively declined, suggesting the loss of the co-transfected cells over time. Interestingly, this decay was accelerated after a second electroporation, suggesting that a specific immune response directed against Min(RA)Gag was mediating this clearance, instead of a direct toxic effect by the construct. Second, since mice cannot get infected with HIV-1, and humanised mice do not have a fully competent immune system, we developed an indirect mouse model to investigate the functionality of the immune response generated after vaccination with Min(RA)Gag-VLPs. This model was based on the inoculation of a Min-expressing tumour cell line derived from B16F10 melanoma cells after VLP immunisation. These Min-expressing cells would act as a surrogate of HIV-1 infected cells by expressing Min proteins on its surface, and functional immune responses could delay tumour growth. B16F10 cells were chosen for their poorly immunogenic profile and because they are resistant to T-cell responses^46^; thus, any effect on tumour growth would be more likely due to a Min-specific antibody-dependent effect. With this model we demonstrated that Min(RA)Gag-VLP-vaccinated animals significantly halted tumour progression compared to Gag-VLP-vaccinated animals. Min(RA)Gag-VLP-vaccinated animals developed anti-Min antibodies that bound to Min on the surface of B16F10Min cells, and also bound to CD16-2 (mFcγRIV) via the Fc, potentially mediating protection. To further support these observations, Min(RA)Gag-VLPs induced a weak anti-Min and anti-Gag cellular immune response.

Taken together, these results show that our engineered fusion-protein generated high-density MinGag-VLPs that induced a potent and functional immune response against a gp41-derived protein, in the absence of adjuvant and at low VLP dose. Finally, VLP-induced antibodies potentially mediate antibody-dependent effector functions through binding to CD16-2. Induction of antibodies with effector function capabilities is relevant, since in the RV114 clinical trial the presence of anti-HIV-1 IgGs that mediated antibody-dependent responses correlated with protection^53^. Further studies will help unveiling the versatility of this VLP platform to present more complex immunogens, such as larger antigens or high ordered structural antigens (full HIV-1 gp160 envelope, for instance) and their ability to induce potent neutralising antibody responses.

## Methods

### Plasmids

The *mingag* fusion protein was generated by fusing a codon-optimised HIV-1 subtype B gp41 miniprotein (“*min*”) containing a fragment of the HR2 domain, the membrane proximal external region (MPER) and the transmembrane domain (TM) (HXB2: 8057-8345 bp)^36^ with a codon-optimised HIV-1 subtype B *gag*_*HXB2*_. A flexible GGGS linker was inserted between the C-terminal end of Min (behind the transmembrane domain) and the N-terminal end of Gag (Fig. 1a). For the production of regular HIV-1 VLPs, a plasmid coding for HIV-1 Gag (HXB2 isolate) and a plasmid coding for a version of the Min antigen containing the intracellular domain of gp41 (HXB2: 8057–8792 bp, Minfull) were also designed^36^. For the generation of MinGag variants, the transmembrane domain of the *mingag* construct was replaced by the transmembrane region of CD44 or an R696A mutated version of the gp41 transmembrane domain (GeneArt). Also, a linker-free construct that joints directly the TM domain of Min with the N-terminal region of Gag was assayed.

Codon-optimised sequences were generated by GeneArt (ThermoFisher Scientific, Waltham, USA) and were cloned into both pcDNA3.1 and pVAX1 vectors (ThermoFisher Scientific) using the *KpnI* and *XhoI* restriction enzymes and the T4-DNA ligase (ThermoFisher Scientific). pcDNA3.1- and pVAX1-derived vectors were used for in vitro VLP production or in vivo DNA immunisation, respectively. Endotoxin-free plasmids were purified with HiSpeed Plasmid Maxi or Mega EF Kits (QIAGEN, Hilden, Germany) and filtered at 0.22 µm before use.

### VLP production, purification, and characterisation

Expi293F cells (ThermoFisher Scientific) were grown in serum-free (SF) and animal-derived component free (ADCF) conditions using Expi293F medium (ThermoFisher Scientific). Cells were transiently transfected with pcDNA3.1-*gag*, pcDNA3.1-*mingag* or pcDNA3.1-*min(RA)gag* encoding vectors or a mix of pcDNA3.1-*minfull* and pcDNA3.1-*gag* plasmids using ExpiFectamine293 Transfection kit and following manufacturer’s protocol (ThermoFisher Scientific). Supernatant was harvested 48 h post-transfection and clarified by centrifugation at 3000 × *g* for 5 min and 0.22 µm filtration. Transfected Expi293F cells were characterised by Fluorescence-Activated Cell Sorting (FACS). Briefly, cells were stained extracellularly with the anti-MPER antibody 10E8 (ARP-12294; NIH HIV Reagent Program, Bethesda, MD, USA) at 1 µg/mL plus a secondary APC-conjugated AffiniPure Goat anti-human IgG (109-136-098; Jackson ImmunoResearch, West Grove, PA, USA) at a 1:500 dilution and intracellularly stained with KC57-PE (6604667; Beckman Coulter, Brea, CA, USA) at a 1:200 dilution. Samples were fixed and permeabilised with Fix&Perm (ThermoFisher Scientific) and analysed using a BD FACSCelesta™ Flow Cytometer (Becton Dickinson (BD), Franklin Lakes, NJ, USA). Gating strategies are displayed in Supplementary Fig. 1.

VLPs were concentrated from cell-cultured supernatant and buffer exchanged to PBS by tangential flow filtration (TFF) with a 300,000 MWCO cassette (Pall Laboratories, Port Washington, NY, USA). Concentrated VLPs were purified by two chromatography steps using: (1) ligand-activated core chromatography HiTrap CaptoCore700 column (Cytiva, Marlborough, MA, USA)^54^, and, (2) anion exchange (AEX) HiTrap Q XL column (Cytiva)^55^. The ÄKTA start chromatography system (Cytiva) was used for protein purification. This purification protocol was compared to sucrose-cushion ultracentrifugation (UC). Finally, VLPs were concentrated at 1 mg/mL of total protein and sterilised by filtration using PVDF 0.22 µm filter (Merck & Co., Kenilworth, NJ, USA).

Purified VLPs were quantified by ELISA (INNOTEST HIV antigen mAb, Fujirebio, Tokyo, Japan) and total protein was quantified using the Pierce BCA Protein Assay (ThermoFisher Scientific). Purified VLPs were routinely characterised by Western Blot (WB) using anti-MPER antibodies 2F5 (AB001; Polymun Scientific, Klosterneuburg, Austria) and 10E8 (NIH HIV Reagent Program) at 4 µg/mL and 1 µg/mL, respectively, and an anti-HIV-1 p24 (subsequently referred as p24) antibody [clone 39/5.4 A] (ab9071; Abcam, Cambridge, UK) at 1 µg/mL. Western blot analysis was performed on TransBlot® Turbo™ Membrane (Bio-Rad, Hercules, CA, USA) using a rabbit polyclonal anti-p15 antibody (ab66951; Abcam) at a 1:1000 dilution and the human antibody 2F5 (Polymun) at 4 µg/mL, for the detection of HIV-1 Gag and MPER, respectively. An IRDye® 680RD-labelled goat anti-rabbit (IgG Fc) (926-68071; LI-COR, Lincoln, NE, USA) at a 1:20,000 dilution and an HRP-labelled goat anti-human IgG (H + L) (109-035-088; Jackson ImmunoResearch) at a 1:20,000 dilution were used as secondary antibodies. Western blot images were obtained in a Chemidoc™ MP Imaging System (Bio-Rad). Precision Plus Protein™ Dual Xtra Prestained Standard (Bio-Rad) was used as a molecular ladder. The band intensity corresponding to Gag, Minfull, and MinGag was calculated with the Image Lab 6 software (Bio-Rad). The ratio of Min (2F5 staining) and Gag (p15 staining) signals were calculated as a relative measure of antigen density in VLPs.

The VLP morphology was assessed by cryogenic electron microscopy (cryo-EM) on a carbon-coated copper grid that was prepared using a Leica EM GP workstation (Leica, Wetzlam, Germany). VLPs were analyzed using a Jeol JEM-2011 electron microscope (JEOL Ltd, Tokyo, Japan), equipped with a CCD 895 USC4000 camera (Gatan, Pleasanton, CA, USA).

### Animal procedures

All animal work was performed at the Centre for Comparative Medicine and Bioimage (CMCiB) under the approval of the Committee on the Ethics of Animal Experimentation of the Germans Trias i Pujol Research Institute (IGTP) and the authorisation of *Generalitat de Catalunya* (codes: 9525 and 9943). All procedures are in accordance with the 3 R principle and prioritise animal welfare.

### In vivo electroporation and bioluminescence

C57BL/6JOlaHsd (Envigo, Horst, Netherlands) mice were intramuscularly injected in the hindlimbs. For the bioluminescence assay, we prepared a 1:1 mix of a firefly luciferase encoding vector (pVAX1-*Fluc*) and pVAX1 empty vector and a 1:1 mix of pVAX1-*Fluc* and pVAX1-*min(RA)gag*. Twenty micrograms of each plasmid mix were injected at the right hindlimb and left hindlimb, respectively. Immediately after each DNA injection, electroporation was performed at the injection site using NEPA21 electroporator (NepaGene, Chiba, Japan). Two different electroporation protocols were used. For the immunisation studies, we used an electroporation protocol optimised for animal welfare consisting of two high-voltage poring pulses (200 V and 0.1 milliseconds (ms) positive pulses with a 20% decay separated by a 10 ms interval) followed by six low-voltage transfer pulses (3 pulses of 60 V, 20 ms and positive polarity with a 20% decay separated by a 50 ms interval, followed by three pulses with the same parameters but inverted polarity). For the in vivo bioluminescence assays, we used an electroporation protocol optimised in house for maximal efficiency and consisted of eight positive pulses of 60 V lasting 20 ms with a 1 s interval.

For in vivo bioluminescence analysis, D-luciferin (150 mg/kg, Biovision, Milpitas, CA) was injected intraperitoneally to mice. Then, mice were anaesthetised using 5% isoflurane at 1 L/min for induction and maintained in the imaging system with 3% isoflurane. Images were taken with 30 s of exposure time and medium binning using an IVIS Lumina III In Vivo Imaging System (Perkin Elmer, Waltham, MA, USA).

### Immunisation

VLP immunogenicity was assessed in C57BL/6JolaHsd (Envigo) mice following two different approaches to assess the effect of combined regimens: (a) a homologous regimen consisting of four doses of purified VLPs (VVVV; 90 ng p24/dose in PBS), or (b) a heterologous regimen consisting of two doses of electroporated DNA (20 µg DNA in PBS) followed by two doses of purified VLPs (DDVV). Purified VLPs were injected subcutaneously into the hock^56^. Each immunisation was performed with a 3-week interval. Experimental groups were Gag-, MinGag- or Min(RA)Gag-immunised animals and controls were pVAX1/PBS or PBS-injected animals.

Before each immunisation, a small blood sample was obtained from the facial vein. Serum was recovered from whole blood by coagulation and centrifugation, and then were heat-inactivated at 56 °C. Spleens were collected at endpoint and disaggregated through a 70 µm mesh (Greiner Bio-One, Kremsmünster, Germany). Splenocytes were cryopreserved in Fetal Bovine Serum (FBS) with 10% dimethyl sulfoxide (DMSO) in liquid nitrogen until further use.

### In vivo protection assay

A C57BL/6JolaHsd mouse model inoculated with a poorly immunogenic, Min-expressing, syngeneic tumour cell line (Supplementary Fig. 7a–c) was employed to functionally assess Min(RA)Gag-VLP-induced immune responses. Briefly, a B16F10 melanoma cell line (ATCC, Manassas, VA, USA) was stably transfected with pcDNA3.1-Min and cells were selected with DMEM supplemented with 2 mg/mL geneticin and 10% heat-inactivated FBS (ThermoFisher Scientific). Geneticin-resistant Min-expressing cells were stained with the 10E8 antibody (NIH HIV Reagent Program) at 1 µg/mL and single-cell sorted with a BD FACSAriaIII Cell Sorter (BD) (Supplementary Fig. 7).

C57BL/6JolaHsd mice were immunised twice with purified Gag-VLPs or MinGag-VLPs. Two weeks after the last immunisation, mice were subcutaneously inoculated with 100,000 B16F10Min-expressing cells in the right flank (Fig. 6a). Tumour length and width was measured periodically with a calliper and tumour volume was calculated as follows^57^: 0.52*(length*(width^2^)). Humane endpoint was reached when tumour size was bigger than 1 cm^3^.

### Production of recombinant proteins

Recombinant Gag and MinTT (MinTT contained a fragment of HR2, the MPER, the gp41 transmembrane domain and the tetanus toxoid (TT) at C-terminus^16,36^) proteins were produced in OneShot™ BL21(DE3) cells (ThermoFisher Scientific) and extracted from inclusion bodies with an 8 M urea buffer. Protein was purified by ion-metal affinity chromatography with 1 mL of Ni^2+^-Sepharose® High Performance beads (Cytiva) and eluted with 0.5 M imidazole in 8 M urea buffer. Protein was concentrated and buffer exchanged to PBS + 0.1% SDS with an Amicon Ultra 15 mL Centrifugal Filter (Merck & Co.)^16^.

### ELISA

Concentration of antibodies against Gag and Min in mouse sera samples was determined by ELISA. Nunc MaxiSorp 96-well plates (ThermoFisher Scientific) were coated with either 50 ng of recombinant Gag or MinTT in PBS or 500 ng of overlapping Min peptides in 0.2 M carbonate/bicarbonate buffer at pH = 9.6 and incubated overnight at 4 °C. After coating, the plate was washed with 1× PBS + 0.05% Tween20 and blocked with 1% BSA + 0.05% Tween20. Then, samples were incubated overnight. Increasing concentrations of mouse monoclonal anti-HIV-1 p24 antibody (clone 39/5.4 A; Abcam) or an anti-gp41 antibody (clone D50, NIH HIV Reagent Program) were used to generate standard curves for Gag and Min, respectively. Results were expressed as p24 or D50 equivalents^16^. Total IgG was determined with a secondary HRP-conjugated AffiniPure Goat anti-mouse IgG (115-036-071; Jackson ImmunoResearch) at a 1:10,000 dilution. IgG subclasses were determined using Biotin-conjugated AffiniPure goat anti-mouse IgG1, IgG2b, IgG2c, and IgG3 antibodies (1106205, 115-065-207, 115-065-208 and 115-065-209, respectively; Jackson ImmunoResearch) at a 1:5,000 dilution and an HRP-conjugated Streptavidin (N100; ThermoFisher Scientific) at a 1:6000 dilution. Finally, after five washes, the assay was developed using o-phenylenediamine (OPD; ThermoFisher Scientific) for 10 min and stopped using 4 N H_2_SO_4_.

### Flow cytometry for the detection of specific humoral responses against Expi293F, B16F10 and B16F10Min cells

Concentration of murine antibodies targeting proteins on the surface of Expi293F, B16F10, or B16F10Min cells was determined by flow cytometry. Cells were incubated with mouse sera samples for 30 min at room temperature (RT), washed with PBS + 10%FBS, and then incubated for 15 min at RT with a secondary AlexaFluor647-labelled goat anti-mouse IgG Fc (115-605-071; Jackson ImmunoResearch). Cells were acquired in a FACSCelesta™ Flow Cytometer (BD) and mean fluorescence intensity (MFI) was analysed with FlowJo_v10.6.1 (BD). To evaluate the specific response induced against Min, the MFI ratio of B16F10Min and B16F10 cells was calculated.

### In vitro Neutralisation assay

Antibody neutralisation capacity of mouse sera was analysed by an in vitro pseudoviral neutralisation assay using four subtype B HIV-1 Env (Bal.01, NL4-3, AC10.0.29, TRO.11) (NIH HIV Reagent Program), an HIV-2/HIV-1 MPER chimaera (7312 A/C1) and an HIV-2 Env control (7312 A) plasmids^58^. Pseudoviruses were generated by co-transfection of Expi293F using Expifectamine293 co-transfected with each Env-expressing plasmid and pSG3Δenv (NIH HIV Reagent Program). Pseudovirus-containing supernatants were harvested 48 h post-transfection^59^. For the neutralisation assay, serum samples were diluted 1/100 in DMEM supplemented with 10% FBS and incubated for 60 min with pseudoviruses at 37 °C. After that, 10,000 TZM-bl cells/well (NIH HIV Reagent Program) were added and cultured for 48 h in the presence of the pseudoviruses/mouse sera mix and 7.5 µg/mL of DEAE-dextran for 48 h. Readout was obtained using BriteLite plus Reporter Gene Assay System (Perkin Elmer)^16,60^ and bioluminescence detected with an Ensight Multimode Plate Reader (Perkin Elmer).

### Binding to CD16-2

Determination of anti-Min antibody binding to mouse CD16-2 (mFcγRIV) receptor was assessed by ELISA. Nunc MaxiSorp plates were coated overnight at 4 °C with 500 ng/well of MinTT in 0.2 M carbonate/bicarbonate buffer at pH = 9.6; background was determined in uncoated wells. Then, plates were blocked for 2 h at RT with 1% BSA and diluted sera samples from immunised mice were added and incubated overnight at 4 °C. After washing, plates were incubated overnight at 4 °C with 1 µg/mL of biotinylated mouse CD16-2 recombinant protein (AcroBiosystems, Newark, DE, USA). CD16-2 binding to Min antibodies was detected using HRP-Streptavidin (ThermoFisher Scientific) for 30 min at RT and TMB substrate. Reaction was stopped with 100 µl of 1 N H_2_SO_4_. Absorbance was read at 450 nm with background subtraction at 620 nm using an Ensight Plate Reader (Perkin Elmer).

### IFN-γ ELISpot assay

Splenocytes from vaccinated animals were seeded at 0.4 × 10^6^ cells/well in ELISpot white PVDF plates (Merck & Co.) precoated with 0.2 µg/well of anti-mouse IFN-γ antibodies (AN18; BioLegend, San Diego, CA, USA) and blocked with 10% FBS-supplemented RPMI (R10). Cells were stimulated overnight at 37 °C and 5% CO_2_ with either a pool of all overlapping Min peptides (Supplementary Fig. 4a; 15 a.a. long overlapped by 11 residues; Covalab, Bron, France) or a pool of 20 overlapping Gag peptides (18 a.a. long overlapped by 9 residues covering p24, p2 and p7) at a concentration of 14 µg/mL per peptide. Concanavalin A (ConA; 7 µg/mL; Merck & Co.) and R10 + 0.5% DMSO were used as positive and negative controls, respectively. IFN-γ secretion was detected with an anti-mIFN-γ biotinylated-mAb (R4-6A2; BioLegend) at a 1:2000 dilution and streptavidin-AP (Mabtech, Cincinnati, OH, USA) and developed with AP Conjugate Substrate Kit (Bio-Rad) at a 1:2000 dilution.

### Statistical analysis

Statistical analyses were performed using Prism 9.0 (GraphPad software Inc., CA, USA) and R-3.6.3 (R Foundation for Statistical Computing). Multiple comparisons were performed using Kruskal–Wallis tests with Dunn’s comparison or Tukey’s multiple comparison test. Unpaired data were compared using a non-parametric Mann–Whitney test. Bioluminescence assay curves were compared using nested mixed effect models and considering time as a categorical factor with a likelihood ratio test. Survival in a Kaplan–Meier table was analysed using a Mantel–Cox test. A two-sided *p* value below 0.05 was considered statistically significant.

### Reporting summary

Further information on research design is available in the Nature Research Reporting Summary linked to this article.

## Supplementary information

Supplemental Figures 1–8 and Supplemental Table 1

REPORTING SUMMARY

## Data Availability

All sequences used are properly described in the material and methods section. Data are available upon request to the authors.

## Code availability

All statistical analyses displayed in Figs. 1–6 were performed with publicly available R packages using R-3.6.3 (R Foundation for Statistical Computing).

## References

[1] Amitai A, Chakraborty AK, Kardar M (2018). The low spike density of HIV may have evolved because of the effects of T helper cell depletion on affinity maturation. PLoS Comput. Biol..

[2] Klein JS, Bjorkman PJ (2010). Few and far between: how HIV may be evading antibody avidity. PLoS Pathog..

[3] Bachmann MF (1993). The influence of antigen organization on B cell responsiveness. Science.

[4] Batista FD, Neuberger MS (1998). Affinity dependence of the B cell response to antigen: a threshold, a ceiling, and the importance of off-rate. Immunity.

[5] Kato Y (2020). Multifaceted effects of antigen valency on B cell response composition and differentiation in vivo. Immunity.

[6] Viant C (2020). Antibody affinity shapes the choice between memory and germinal center B cell fates. Cell.

[7] Heath PT (2021). Safety and efficacy of NVX-CoV2373 Covid-19 vaccine. N. Engl. J. Med..

[8] Brouwer PJM (2019). Enhancing and shaping the immunogenicity of native-like HIV-1 envelope trimers with a two-component protein nanoparticle. Nat. Commun..

[9] Hardy GJ (2014). HIV-1 antibodies and vaccine antigen selectively interact with lipid domains. Biochim. Biophys. Acta.

[10] Jardine JG (2016). HIV-1 broadly neutralizing antibody precursor B cells revealed by germline-targeting immunogen. Science.

[11] Krebs SJ (2014). Multimeric scaffolds displaying the HIV-1 envelope MPER induce MPER-specific antibodies and cross-neutralizing antibodies when co-immunized with gp160 DNA. PLoS ONE.

[12] Sliepen K (2015). Presenting native-like HIV-1 envelope trimers on ferritin nanoparticles improves their immunogenicity. Retrovirology.

[13] Tokatlian T (2018). Enhancing humoral responses against HIV envelope trimers via nanoparticle delivery with stabilized synthetic liposomes. Sci. Rep..

[14] Wieczorek L (2015). Comparable antigenicity and immunogenicity of oligomeric forms of a novel, acute HIV-1 subtype C gp145 envelope for use in preclinical and clinical vaccine research. J. Virol..

[15] Ingale J (2016). High-density array of well-ordered HIV-1 spikes on synthetic liposomal nanoparticles efficiently activate B cells. Cell Rep..

[16] Molinos-Albert LM (2017). Proteoliposomal formulations of an HIV-1 gp41-based miniprotein elicit a lipid-dependent immunodominant response overlapping the 2F5 binding motif. Sci. Rep..

[17] Zhang P (2021). A multiclade env–gag VLP mRNA vaccine elicits tier-2 HIV-1-neutralizing antibodies and reduces the risk of heterologous SHIV infection in macaques. Nat. Med..

[18] Cervera L (2019). Production of HIV-1-based virus-like particles for vaccination: achievements and limits. Appl. Microbiol. Biotechnol..

[19] Carlson L-A (2008). Three-dimensional analysis of budding sites and released virus suggests a revised model for HIV-1 morphogenesis. Cell Host Microbe.

[20] Lavado-García J (2021). Characterization of HIV-1 virus-like particles and determination of Gag stoichiometry for different production platforms. Biotechnol. Bioeng..

[21] Buchbinder SP (2017). Immunogenicity of a novel Clade B HIV-1 vaccine combination: results of phase 1 randomized placebo controlled trial of an HIV-1 GM-CSF-expressing DNA prime with a modified vaccinia Ankara vaccine boost in healthy HIV-1 uninfected adults. PLoS ONE.

[22] Perdiguero B (2019). A novel MVA-based HIV vaccine candidate (MVA-gp145-GPN) co-expressing clade C membrane-bound trimeric gp145 Env and Gag-induced virus-like particles (VLPs) triggered broad and multifunctional HIV-1-specific T cell and antibody responses. Viruses.

[23] Chapman R (2020). Immunogenicity of HIV-1 vaccines expressing chimeric envelope glycoproteins on the surface of Pr55 Gag virus-like particles. Vaccines.

[24] Gonelli CA (2021). Immunogenicity of HIV-1-based virus-like particles with increased incorporation and stability of membrane-bound Env. Vaccines.

[25] Beltran-Pavez C (2018). Guiding the humoral response against HIV-1 toward a MPER adjacent region by immunization with a VLP-formulated antibody-selected envelope variant. PLoS One.

[26] Crooks ET (2017). Effects of partially dismantling the CD4 binding site glycan fence of HIV-1 envelope glycoprotein trimers on neutralizing antibody induction. Virology.

[27] Tohidi F, Sadat SM, Bolhassani A, Yaghobi R, Larijani MS (2019). Induction of a robust humoral response using HIV-1 VLP(MPER-V3) as a novel candidate vaccine in BALB/c mice. Curr. HIV Res..

[28] Xiao P (2021). Parainfluenza virus 5 priming followed by SIV/HIV virus-like-particle boosting induces potent and durable immune responses in nonhuman primates. Front. Immunol..

[29] Storcksdieck genannt Bonsmann M, Niezold T, Hannaman D, Überla K, Tenbusch M (2016). The improved antibody response against HIV-1 after a vaccination based on intrastructural help is complemented by functional CD8+ T cell responses. Vaccine.

[30] Huang X (2017). In vivo electroporation in DNA-VLP prime-boost preferentially enhances HIV-1 envelope-specific IgG2a, neutralizing antibody and CD8 T cell responses. Vaccine.

[31] Storcksdieck genannt Bonsmann M (2015). Enhancing the quality of antibodies to HIV-1 envelope by GagPol-specific Th cells. J. Immunol..

[32] Crooks ET (2015). Vaccine-elicited tier 2 HIV-1 neutralizing antibodies bind to quaternary epitopes involving glycan-deficient patches proximal to the CD4 binding site. PLoS Pathog..

[33] Escolano A (2019). Immunization expands B cells specific to HIV-1 V3 glycan in mice and macaques. Nature.

[34] Stano A (2017). Dense array of spikes on HIV-1 virion particles. J. Virol..

[35] Molinos-Albert LM, Clotet B, Blanco J, Carrillo J (2017). Immunologic insights on the membrane proximal external region: a major human immunodeficiency virus type-1 vaccine target. Front. Immunol..

[36] Molinos-Albert LM (2014). Anti-MPER antibodies with heterogeneous neutralization capacity are detectable in most untreated HIV-1 infected individuals. Retrovirology.

[37] Oliferenko S (1999). Analysis of CD44-containing lipid rafts: recruitment of annexin II and stabilization by the actin cytoskeleton. J. Cell Biol..

[38] Kim JH, Hartley TL, Curran AR, Engelman DM (2009). Molecular dynamics studies of the transmembrane domain of gp41 from HIV-1. Biochim. Biophys. Acta..

[39] Excler J-L, Kim JH (2019). Novel prime-boost vaccine strategies against HIV-1. Expert Rev. Vaccines.

[40] Atmar RL (2022). Homologous and heterologous Covid-19 booster vaccinations. N. Engl. J. Med..

[41] Lux A, Nimmerjahn F (2013). Of mice and men: the need for humanized mouse models to study human IgG activity in vivo. J. Clin. Immunol..

[42] Watzl C, Long EO (2010). Signal transduction during activation and inhibition of natural killer cells. Curr. Protoc. Immunol..

[43] Collins AM (2016). IgG subclass co-expression brings harmony to the quartet model of murine IgG function. Immunol. Cell Biol..

[44] Verkoczy L (2011). Rescue of HIV-1 broad neutralizing antibody-expressing B cells in 2F5 VH x VL knockin mice reveals multiple tolerance controls. J. Immunol..

[45] Verkoczy L (2013). Induction of HIV-1 broad neutralizing antibodies in 2F5 knock-in mice: selection against membrane proximal external region-associated autoreactivity limits T-dependent responses. J. Immunol..

[46] Wang J, Saffold S, Cao X, Krauss J, Chen W (1998). Eliciting T cell immunity against poorly immunogenic tumors by immunization with dendritic cell-tumor fusion vaccines. J. Immunol..

[47] Graham BS, Gilman MSA, McLellan JS (2019). Structure-based vaccine antigen design. Annu. Rev. Med..

[48] Elsayed H (2018). Intrastructural help: harnessing T helper cells induced by licensed vaccines for improvement of HIV Env antibody responses to virus-like particle vaccines. J. Virol..

[49] Moyer TJ, Zmolek AC, Irvine DJ (2016). Beyond antigens and adjuvants: formulating future vaccines. J. Clin. Invest..

[50] Borobia AM (2021). Immunogenicity and reactogenicity of BNT162b2 booster in ChAdOx1-S-primed participants (CombiVacS): a multicentre, open-label, randomised, controlled, phase 2 trial. Lancet (Lond., Engl.).

[51] Normark J (2021). Heterologous ChAdOx1 nCoV-19 and mRNA-1273 vaccination. N. Engl. J. Med..

[52] Watanabe H, Numata K, Ito T, Takagi K, Matsukawa A (2004). Innate immune response in Th1- and Th2-dominant mouse strains. Shock.

[53] Rerks-Ngarm S (2009). Vaccination with ALVAC and AIDSVAX to prevent HIV-1 infection in Thailand. N. Engl. J. Med..

[54] Weigel T (2014). A flow-through chromatography process for influenza A and B virus purification. J. Virol. Methods.

[55] Steppert P (2016). Purification of HIV-1 gag virus-like particles and separation of other extracellular particles. J. Chromatogr. A.

[56] Kamala T (2007). Hock immunization: a humane alternative to mouse footpad injections. J. Immunol. Methods.

[57] Mei K-C, Bai J, Lorrio S, Wang JT-W, Al-Jamal KT (2016). Investigating the effect of tumor vascularization on magnetic targeting in vivo using retrospective design of experiment. Biomaterials.

[58] Montefiori DC (2009). Measuring HIV neutralization in a luciferase reporter gene assay. Methods Mol. Biol..

[59] Sánchez-Palomino S (2011). A cell-to-cell HIV transfer assay identifies humoral responses with broad neutralization activity. Vaccine.

[60] Li M (2005). Human immunodeficiency virus type 1 env clones from acute and early subtype B infections for standardized assessments of vaccine-elicited neutralizing antibodies. J. Virol..

