# Supplementary material for: An engineered HIV-1 Gag-based VLP displaying high antigen density induces strong antibody-dependent functional immune responses
Source: NPJ Vaccines. 2023 Apr 6;8:51. doi: 10.1038/s41541-023-00648-4 (PMC10077320; doi:10.1038/s41541-023-00648-4)
Supplement: Supplementary file 1 — Supplemental Figures 1–8 and Supplemental Table 1 [file 41541_2023_648_MOESM1_ESM.pdf]

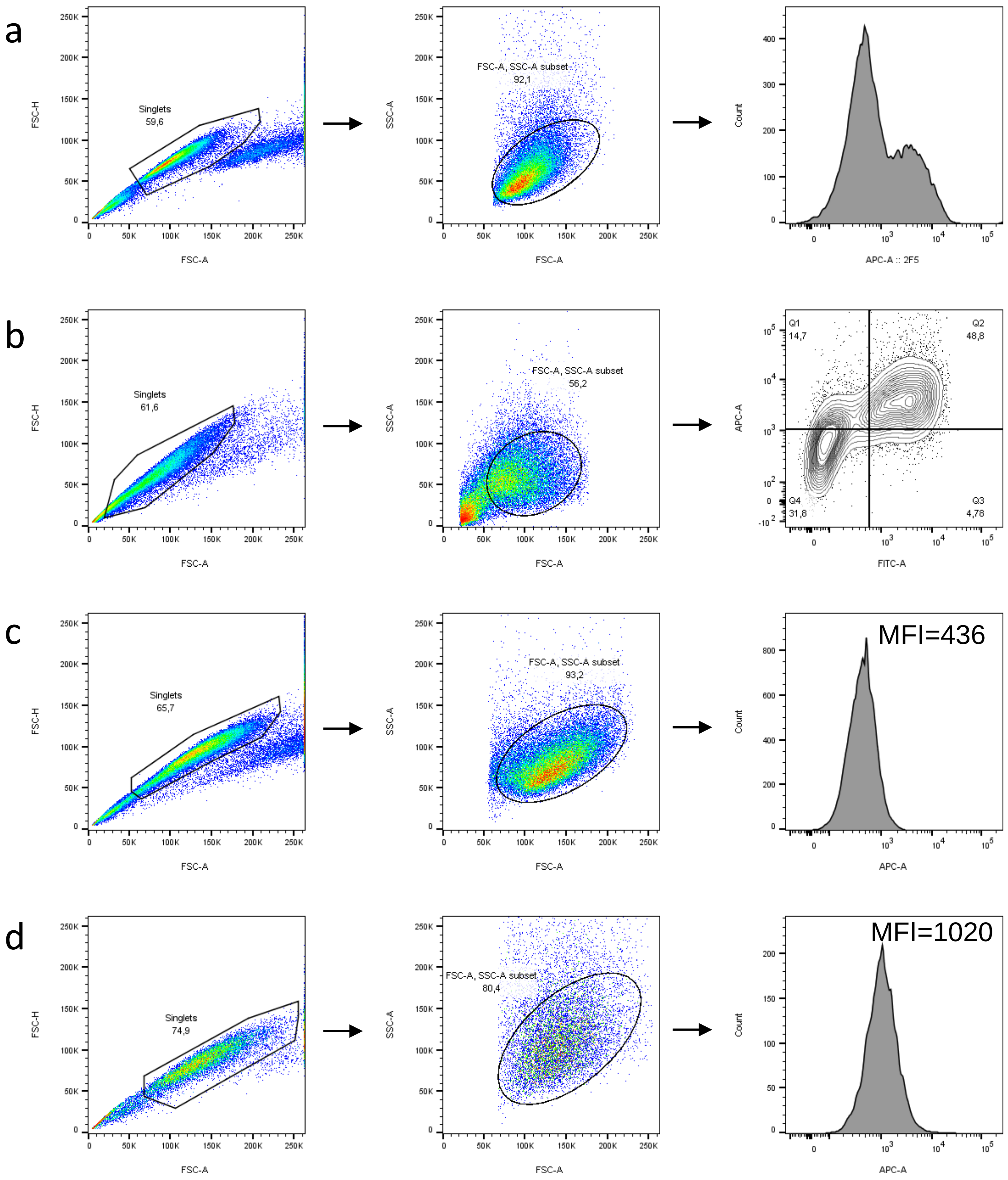

Supplementary Figure 1. **Gating strategies in Flow cytometry panels**. The first plot (left) displays singlets in FSC-A (X-axis) and FSC-H (Y-axis). The second plot (middle) displays FSC-A (X-axis) and SSC-A (Y-axis). The third panel shows an histogram of APC-A. **a)** Dot plots from B16F10Min cells (Supplementary Figure 2b and 2c). **b)** Dot plots of Expi293-transfected and VLP-producing cells (Figures 1b, 2a and 2b). **c)** Dot plots of anti-Expi293F antibodies in mouse serum of VLP-vaccinated animals (Figure 3c and Supplementary Figure 7c). **d)** Dot plots corresponding to the analysis of antibodies against Min protein expressed on the surface of B16F10Min cells. Results in Figure 6e are calculated as the ratio of the mean fluorescence intensity (MFI) on the APC channel of B16F10Min and B16F10 cells.

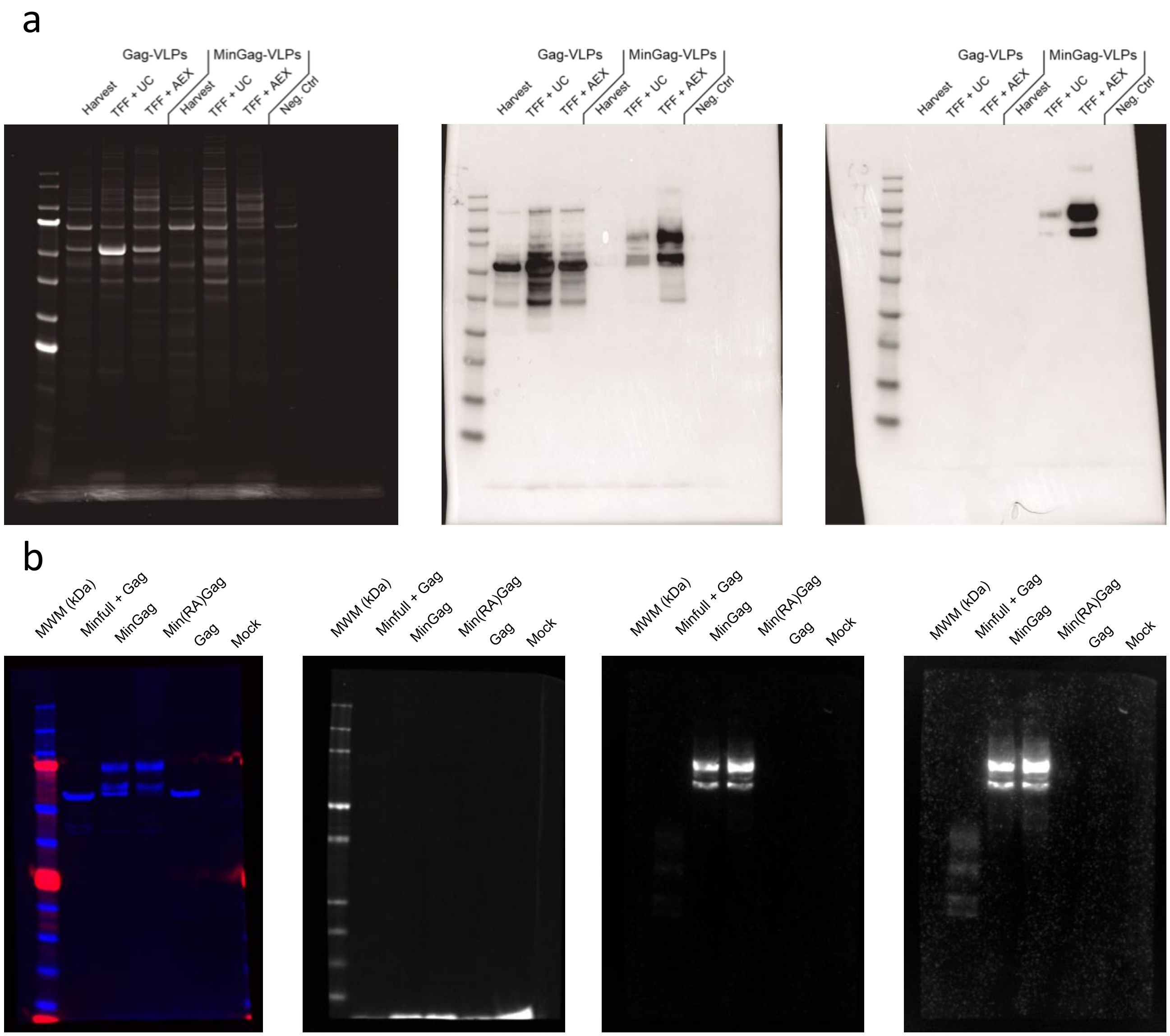

Supplementary Figure 2. **Unprocessed Coomassie blue gels and Western Blots**. **a)** Characterisation of HIV-1 Gag-VLPs and MinGag-VLPs from harvested supernatants and after purification with either sucrose-cushion ultracentrifugation (TFF + UC) or anion exchange chromatography (TFF + AEX) by Coomassie blue staining (left), anti-p24 WB (middle) and anti-2F5 WB (right). **b)** Western Blot analysis of the same gel loaded with VLPs purified from supernatant of Expi293 cells transfected with Minfull + Gag, MinGag or Min(RA)Gag. First panel: anti-p15 antibody. Second panel: colorimetric ladder for chemiluminescence. Third panel: 2F5 antibody (anti-MPER). Fourth panel: 2F5 antibody (anti-MPER) with brightness (+40%) and contrast (-40%) adjustment.

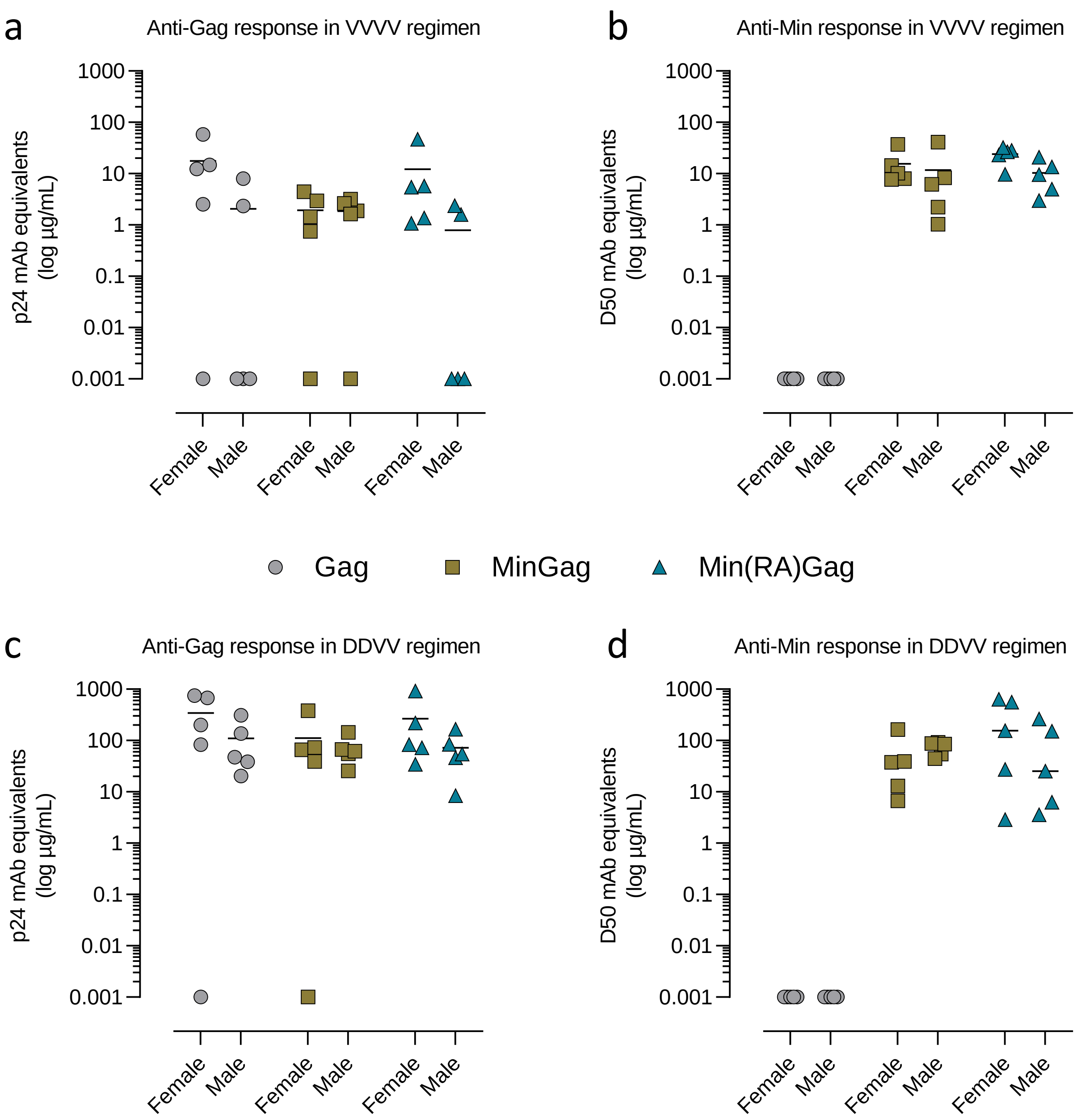

Supplementary Figure 3. **Sex-bias analysis of anti-Gag and anti-Min IgG concentration in the serum of immunised mice.** Mice were immunised with Gag-VLPs (grey rounds, n=10), MinGag-VLPs (gold squares, n=10) or Min(RA)Gag-VLPs (blue triangles, n=10) with either a homologous (VVVV) or heterologous (DDVV) regimen with a 3-week interval between doses. Serum was obtained at week 12. All groups were sex-balanced (50% females). **a)** Anti-Gag responses of animals immunised with a homologous regimen. **b)** Anti-Min responses of mice immunised with a homologous regimen. **c)** Anti-Gag responses of animals immunised with a heterologous regimen. **d)** Anti-Min responses of mice immunised with a heterologous regimen. Data represented as mean±SD. No significant differences were found between males and females using a Kruskal-Wallis test.

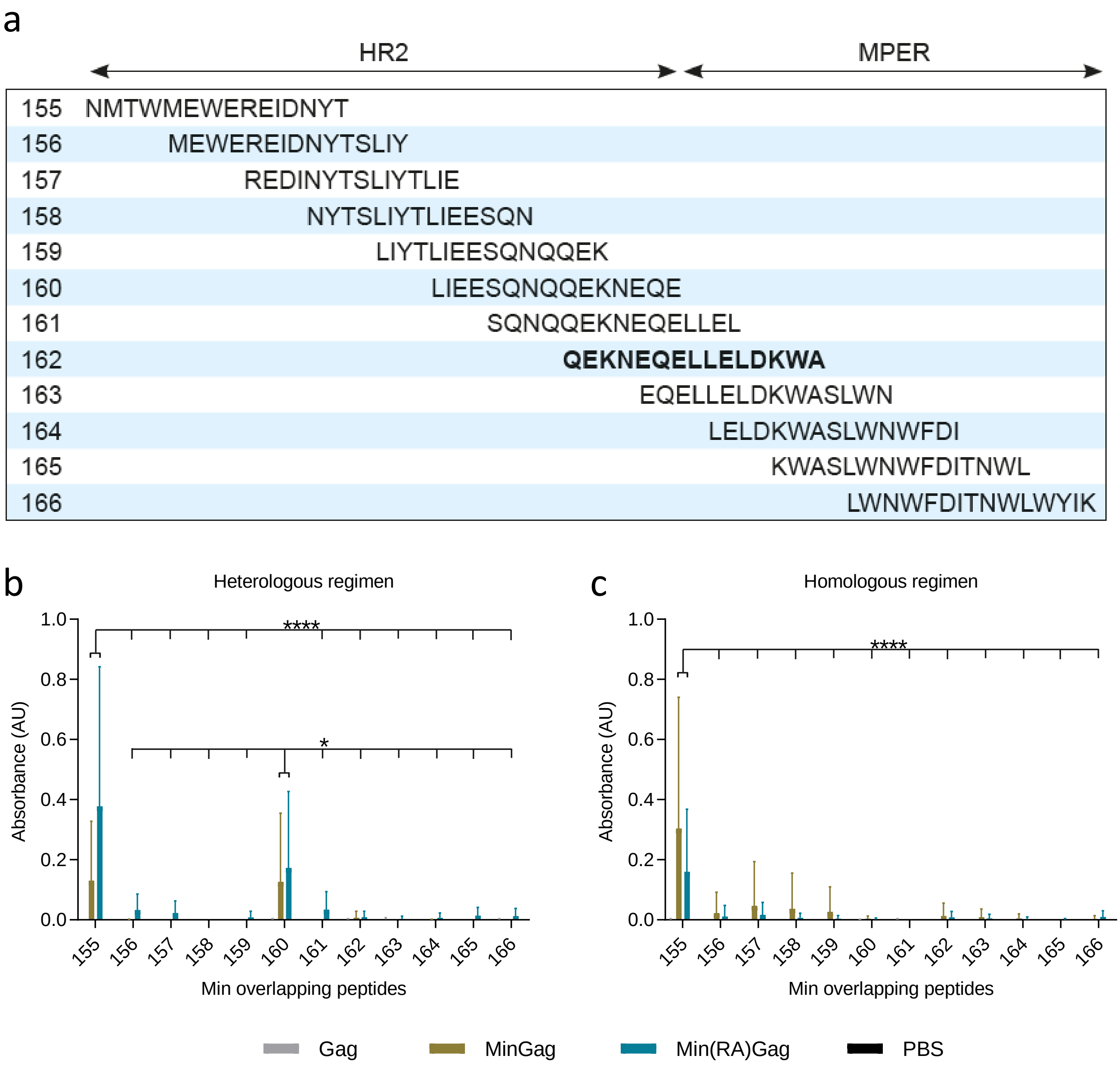

Supplementary Figure 4. **Mapping of the main Min linear epitopes targeted by VLP-induced IgGs.** Mice were immunised with Gag-VLPs (grey, n=10), MinGag-VLPs (gold, n=10) or Min(RA)Gag-VLPs (blue, n=10) with either a homologous (VVVV) or heterologous (DDVV) regimen with a 3-week interval between doses. Serum was obtained at week 12. **a)** Scheme of Min OLPs. **b)** Mapping of the anti-Min response in the heterologous regimen. **c)** Mapping of the anti-Min response in the homologous regimen. Significant differences were found using a Kruskal-Wallis test with Tukey's correction for multiple comparisons (*p<0.05, ****p<0.0001).

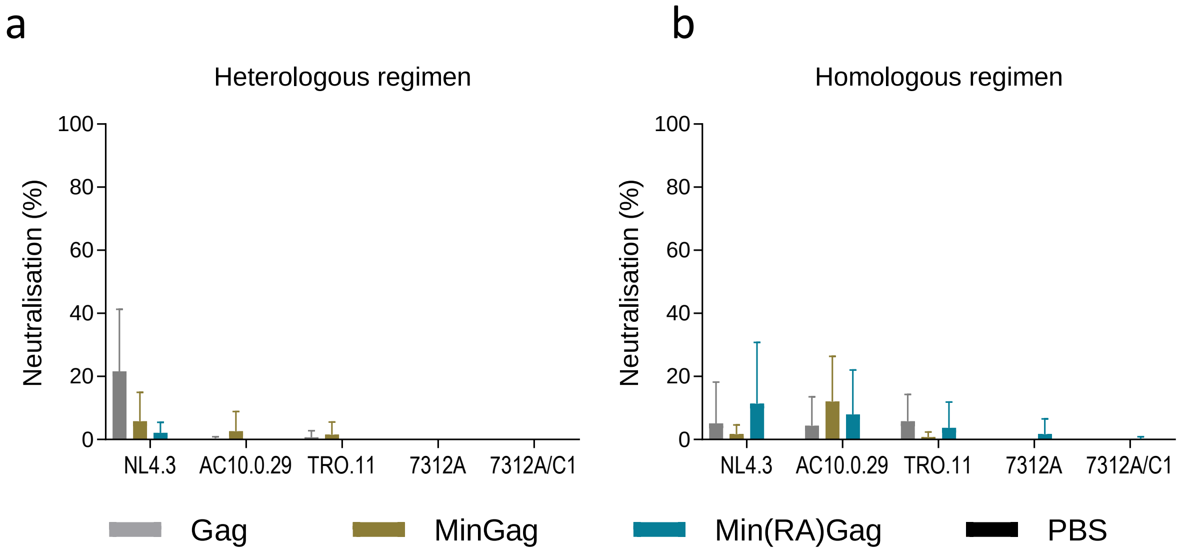

Supplementary Figure 5. **In vitro pseudovirus neutralization assay of serum samples from VLP-immunised mice**, Neutralisation assay was performed using a panel of 3 subtype B HIV-1 pseudoviruses (Tier-1A: NL4-3, Tier1B: Bal.01, Tier-2: TRO.11), a chimaeric HIV-2 with the HIV-1 subtype B MPER (7312A/C1) and its control (7312A). Samples were collected at week 12 after immunisation. **a)** VLP-vaccinated mice using a homologous (VVVV) regimen. **b)** VLP-immunised mice using a heterologous (DDVV) regimen.

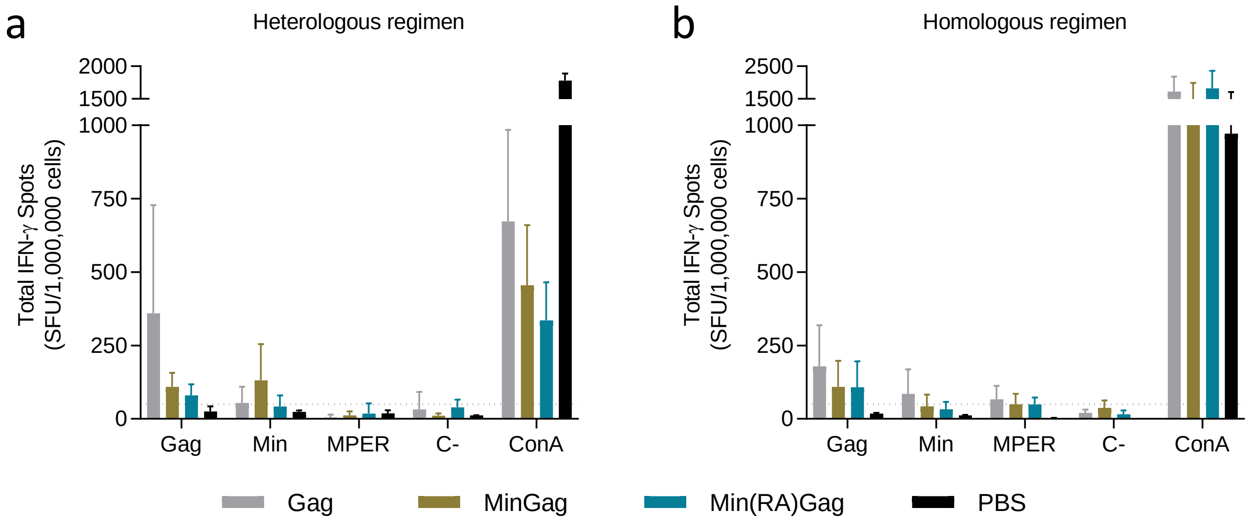

Supplementary Figure 6. **Analysis of cellular immune responses against Gag, Min and MPER**. Splenocytes collected at weeek 12 from C57Bl/6JOlaHsd mice immunised with Gag-VLPs (grey), MinGag-VLPs (gold), Min(RA)Gag-VLPs (blue) and pVAX1/PBS or PBS controls (black) were stimulated *in vitro* with two pools of peptides covering Gag and Min proteins, and with an MPER peptide. Unestimulated splenocytes were used as a negative control (C-) and Concanavalin A (ConA) stimulated splenocytes were used as positive controls. **a)** Splenocytes from mice immunised by a heterologous DNA/VLP regimen of Gag-VLPs (n=8), MinGag-VLPs (n=4), Min(RA)Gag-VLPs (n=3). **b)** Splenocytes from mice immunised by a heterologous DNA/VLP regimen of Gag-VLPs (n=7), MinGag-VLPs (n=7), Min(RA)Gag-VLPs (n=2). No significant differences were found between groups using a Kruskal-Wallis test.

a

Step	Procedure
Transfection	Transfection of pcDNA3.1-*min(tm)* with X-tremeGENE
Selection	Culturing of B16F10Min cells with Geneticin
Sorting	Single-cell sorting of 2F5+ events with a FACSAryaII
Characterisation	Long-term expression of Min (10E8-binding) without antibiotic

b

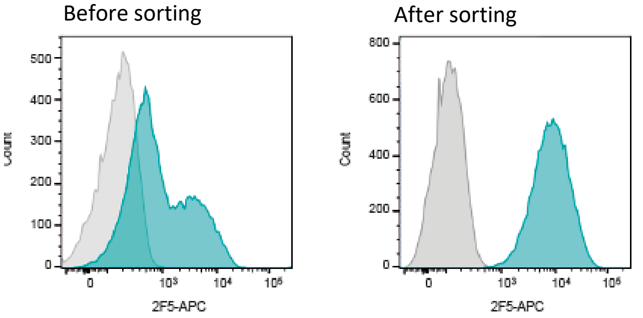

c

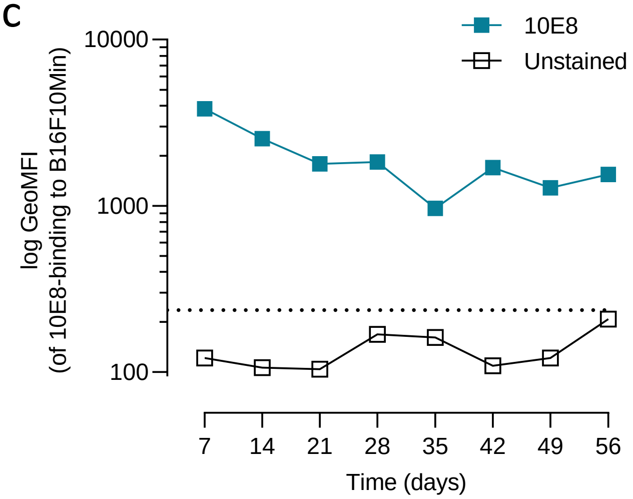

Supplementary Figure 7. **Development of a melanoma B16F10 cell line that stably expresses Min at the cell surface**. **a)** Pipeline for the development of B16F10Min cells. **b)** Histograms showing 2F5-staining of B16F10 (grey) and B16F10Min (blue) cells before (left) and after single cell sorting (right). **c)** Min stability and antigenicity on the Surface of a single-cell sorted B16F10Min clone in absence of selection antibiotic (neomycin). Data represented as Geo-MFI. Dotted line shows the positivity threshold.

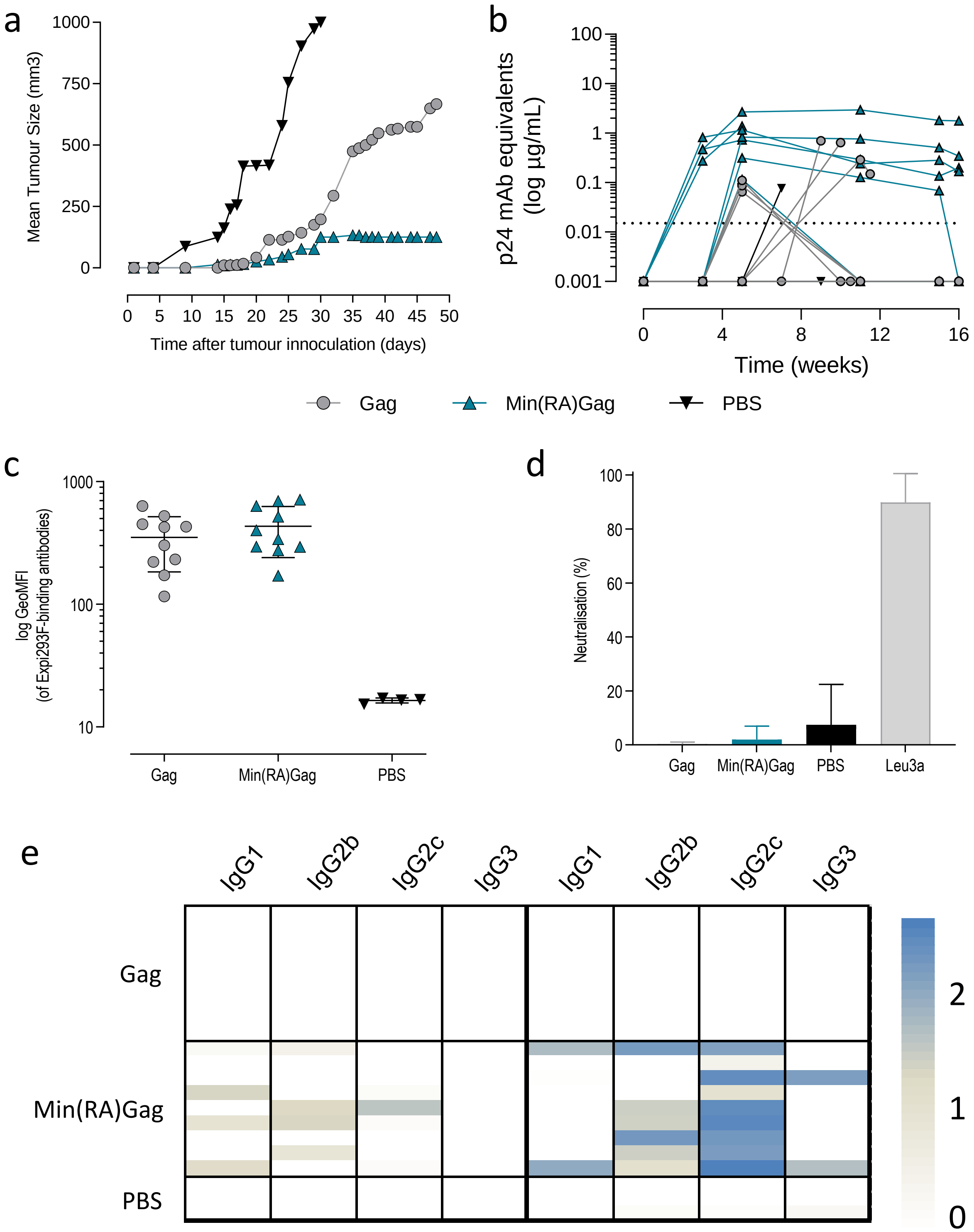

Supplementary Figure 8. **B16F10Min tumour progression and immune profiling of VLP-vaccinated and tumour-inoculated mice.** Mice were vaccinated with 2 doses of Gag-VLPs (grey rounds, n=9), Min(RA)Gag-VLPs (n=8) or PBS (n=4) and inoculated with 100.000 B16F10Min cells. **a)** Tumour growth calculated as 0.52 x length x width^2. Animals were followed-up for 80 days and euthanised once tumour reached a size bigger than 1,000 mm^3. **b)** Follow-up of the concentration of anti-Gag IgG determined by ELISA. **c)** Humoral response against Expi293F VLP-producing cells' host proteins in serum from week 5 determined by FACS. **d)** *In vitro* Bal.01 pseudovirus neutralisation assay with mouse sera collected at endpoint. **e)** Heatmap of the predominant murine IgG subclasses for anti-Gag and anti-Min antibodies in the VLP-immunised and tumour-inoculated mice.

Supplementary Table 1. **Concentrations, yields and purity ratios of Gag-VLPs and MinGag-VLPs produced in Expi293F cells and purified by TFF + UC (top table) or by TFF + AEX (bottom table).**

**Ultracentrifugation**	Supernatant		Final product		Ratio p24:prot			Recovery
Concentration (µg/mL)	p24	Protein	p24	Protein	Initial (%)	Final (%)	Increase	(%)
Gag-VLPs	1,25	3456,89	142,29	4941,53	0,04	2,88	79,79	50,69
MinGag-VLPs	0,10	2190,03	9,58	5591,40	0,00	0,17	36,52	41,43

**Chromatography**	Supernatant		Final product		Ratio p24:prot			Recovery
Concentration (µg/mL)	p24	Protein	p24	Protein	Initial (%)	Final (%)	Increase	(%)
Gag-VLPs	1,48	2748,30	19,77	1399,31	0,05	1,41	26,28	5,95
MinGag-VLPs	0,04	2089,74	4,98	804,91	0,00	0,62	317,86	13,60
Min(RA)Gag-VLPs	0,08	1461,18	12,35	520,68	0,01	2,37	431,02	8,53
