# Supplementary material for: An engineered HIV-1 Gag-based VLP displaying high antigen density induces strong antibody-dependent functional immune responses
Source: NPJ Vaccines. 2023 Apr 6;8:51. doi: 10.1038/s41541-023-00648-4 (PMC10077320; doi:10.1038/s41541-023-00648-4)
Supplement: Supplementary file 2 — REPORTING SUMMARY [file 41541_2023_648_MOESM2_ESM.pdf]

nature portfolio

Corresponding author(s): Julià Blanco
Last updated by author(s): Dec 29, 2022

Reporting Summary

Nature Portfolio wishes to improve the reproducibility of the work that we publish. This form provides structure for consistency and transparency in reporting. For further information on Nature Portfolio policies, see our Editorial Policies and the Editorial Policy Checklist.

Statistics

For all statistical analyses, confirm that the following items are present in the figure legend, table legend, main text, or Methods section.

n/a	Confirmed	
☐	☒	The exact sample size (*n*) for each experimental group/condition, given as a discrete number and unit of measurement
☐	☒	A statement on whether measurements were taken from distinct samples or whether the same sample was measured repeatedly
☐	☒	The statistical test(s) used AND whether they are one- or two-sided<br>*Only common tests should be described solely by name; describe more complex techniques in the Methods section.*
☒	☐	A description of all covariates tested
☐	☒	A description of any assumptions or corrections, such as tests of normality and adjustment for multiple comparisons
☐	☒	A full description of the statistical parameters including central tendency (e.g. means) or other basic estimates (e.g. regression coefficient) AND variation (e.g. standard deviation) or associated estimates of uncertainty (e.g. confidence intervals)
☐	☒	For null hypothesis testing, the test statistic (e.g. F, t, r) with confidence intervals, effect sizes, degrees of freedom and P value noted<br>*Give P values as exact values whenever suitable.*
☒	☐	For Bayesian analysis, information on the choice of priors and Markov chain Monte Carlo settings
☒	☐	For hierarchical and complex designs, identification of the appropriate level for tests and full reporting of outcomes
☒	☐	Estimates of effect sizes (e.g. Cohen's *d*, Pearson's *r*), indicating how they were calculated

Our web collection on statistics for biologists contains articles on many of the points above.

Software and code

Policy information about availability of computer code

Data collection	n/a
Data analysis	Statistical analyses were performed using Prism 9.0 (GraphPad software Inc., CA, USA) and R-3.6.3 (R Foundation for Statistical Computing). Multiple comparisons were performed using Kruskal-Wallis tests with Dunn’s comparison or Tukey’s multiple comparison test. Unpaired data was compared using a non-parametric Mann-Whitney test. Bioluminescence assay curves were compared using nested mixed effect models and considering time as a categorical factor with a likelihood ratio test. Survival in a Kaplan-Meier table was analyzed using a Mantel-Cox test. A two-sided p-value below 0.05 was considered statistically significant.

For manuscripts utilizing custom algorithms or software that are central to the research but not yet described in published literature, software must be made available to editors and reviewers. We strongly encourage code deposition in a community repository (e.g. GitHub). See the Nature Portfolio guidelines for submitting code & software for further information.

Data

Policy information about availability of data

All manuscripts must include a data availability statement. This statement should provide the following information, where applicable:

- Accession codes, unique identifiers, or web links for publicly available datasets
- A description of any restrictions on data availability
- For clinical datasets or third party data, please ensure that the statement adheres to our policy

All sequences used are properly described in the material and methods section. Data is available upon request to the authors.

Human research participants

Policy information about studies involving human research participants and Sex and Gender in Research.

Reporting on sex and gender	n/a
Population characteristics	n/a
Recruitment	n/a
Ethics oversight	n/a

Note that full information on the approval of the study protocol must also be provided in the manuscript.

Field-specific reporting

Please select the one below that is the best fit for your research. If you are not sure, read the appropriate sections before making your selection.

☒ Life sciences ☐ Behavioural & social sciences ☐ Ecological, evolutionary & environmental sciences

For a reference copy of the document with all sections, see nature.com/documents/nr-reporting-summary-flat.pdf

Life sciences study design

All studies must disclose on these points even when the disclosure is negative.

Sample size	To determine the sample size, a statistical power analysis was performed using the G-POWER software. Based on data published in "Castle, J. C., et al. (2012). Cancer Research, 72(5), 1081–1091" the means and standard deviations of treated and control groups were estimated. The sample size necessary to reach a statistical power of 80% was n=10 per group.
Data exclusions	No data exclusion was performed.
Replication	For immunological studies, some samples were retested to confirm the results.
Randomization	The cages of the animals used in this study were randomized upon arrival. Each animal within the same cage recieved the same treatment.
Blinding	Tumour growth measurment was performed blindly. The rest of the experiments were perfromed in an unblinded fashion, since the results are objective and not susceptible to change by personal biases.

Reporting for specific materials, systems and methods

We require information from authors about some types of materials, experimental systems and methods used in many studies. Here, indicate whether each material, system or method listed is relevant to your study. If you are not sure if a list item applies to your research, read the appropriate section before selecting a response.

Materials & experimental systems

n/a	Involved in the study
☐	☒ Antibodies
☐	☒ Eukaryotic cell lines
☐	☐ Palaeontology and archaeology
☐	☒ Animals and other organisms
☐	☐ Clinical data
☐	☐ Dual use research of concern

Methods

n/a	Involved in the study
☐	☐ ChIP-seq
☐	☒ Flow cytometry
☐	☐ MRI-based neuroimaging

Antibodies

Antibodies used

Anti-MPER antibody clone 2F5 (Polymun Scientific, AB001),
anti-MPER antibody clone 10E8 (NIH HIV Reagent Program, ARP-12294),
anti-MPER antibody clone D50 (NIH HIV Reagent Program, ARP-11393),
anti-HIV-1 p24 clone 39/5.4A (abcam, ab9071),
anti-HIV-1 p15 polyclonal antibody (abcam, ab66951),
anti-mouse IFN-g clone AN18 (Biolegend, 517901),
FITC-conjugated anti-HIV-1 Gag clone KC57 (Beckman Coulter, 6604667),
AlexaFluor647-conjugated goat anti-mouse IgG Fc (Jackson ImmunoResearch, 115-605-071),
APC-conjugated goat anti-human IgG Fc (109-136-098),
IRDye 680RD-labelled goat anti-rabbit (IgG Fc) (LICOR, 926-68071),
HRP-labelled goat anti-human IgG Fc (Jackson ImmunoResearch, 109-036-098),
biotin-conjugated AffiniPure goat anti-mouse IgG1, IgG2b, IgG2c and IgG3 antibodies (Jackson ImmunoResearch, 115-065-205, 115-065-207, 115-065-208, 115-065-209, respectively),
biotin-conjugated anti-mouse IFN-g clone R4-6A2 (Biolegend, 505701),
HRP-conjugated Streptavidin (ThermoFisher Scientific, N100),
AP-conjugated Streptavidin (Mabtech, 3310-8).

Validation

All antibodies were tested and titrated in-lab.

Eukaryotic cell lines

Policy information about cell lines and Sex and Gender in Research

Cell line source(s)

Expi293F cells (ThermoFisher Scientific), B16F10 cells (ATCC), TZM-bl cells NIH HIV Reagent Program)

Authentication

The cells used in this manuscript were authenticated by the providers.

Mycoplasma contamination

All cel lines were routinely tested for mycoplasma contamination and only negative cells were used.

Commonly misidentified lines
(See ICLAC register)

n/a

Palaeontology and Archaeology

Specimen provenance

n/a

Specimen deposition

n/a

Dating methods

n/a

☐ Tick this box to confirm that the raw and calibrated dates are available in the paper or in Supplementary Information.

Ethics oversight

n/a

Note that full information on the approval of the study protocol must also be provided in the manuscript.

Animals and other research organisms

Policy information about studies involving animals; ARRIVE guidelines recommended for reporting animal research, and Sex and Gender in Research

Laboratory animals

C57BL/6JOlaHsd

Wild animals

n/a

Reporting on sex	In this manuscript we used mice from both sexes (50% females/50% males). We performed a sex-based analyses and no significant differences were found in the parametres analysed (Supplementary Figure 2).
Field-collected samples	n/a
Ethics oversight	All animal work was performed at the Centre for Comparative Medicine and Bioimage (CMCiB) under the approval of the Committee on the Ethics of Animal Experimentation of the Germans Trias i Pujol Research Institute (IGTP) and the authorisation of Generalitat de Catalunya (codes: 9525 and 9943). All procedures are in accordance with the 3R principle and prioritise animal welfare.

Note that full information on the approval of the study protocol must also be provided in the manuscript.

Clinical data

Policy information about clinical studies

All manuscripts should comply with the ICMJE guidelines for publication of clinical research and a completed CONSORT checklist must be included with all submissions.

Clinical trial registration	n/a
Study protocol	n/a
Data collection	n/a
Outcomes	n/a

Dual use research of concern

Policy information about dual use research of concern

Hazards

Could the accidental, deliberate or reckless misuse of agents or technologies generated in the work, or the application of information presented in the manuscript, pose a threat to:

No	Yes	
☒	☐	Public health
☒	☐	National security
☒	☐	Crops and/or livestock
☒	☐	Ecosystems
☒	☐	Any other significant area

Experiments of concern

Does the work involve any of these experiments of concern:

No	Yes	
☒	☐	Demonstrate how to render a vaccine ineffective
☒	☐	Confer resistance to therapeutically useful antibiotics or antiviral agents
☒	☐	Enhance the virulence of a pathogen or render a nonpathogen virulent
☒	☐	Increase transmissibility of a pathogen
☒	☐	Alter the host range of a pathogen
☒	☐	Enable evasion of diagnostic/detection modalities
☒	☐	Enable the weaponization of a biological agent or toxin
☒	☐	Any other potentially harmful combination of experiments and agents

ChIP-seq

Data deposition

☐ Confirm that both raw and final processed data have been deposited in a public database such as GEO.

☐ Confirm that you have deposited or provided access to graph files (e.g. BED files) for the called peaks.

Data access links<br>*May remain private before publication.*	n/a

Files in database submission	n/a
Genome browser session (e.g. UCSC)	n/a

Methodology

Replicates	n/a
Sequencing depth	n/a
Antibodies	n/a
Peak calling parameters	n/a
Data quality	n/a
Software	n/a

Flow Cytometry

Plots

Confirm that:

☒ The axis labels state the marker and fluorochrome used (e.g. CD4-FITC).

☒ The axis scales are clearly visible. Include numbers along axes only for bottom left plot of group (a 'group' is an analysis of identical markers).

☒ All plots are contour plots with outliers or pseudocolor plots.

☒ A numerical value for number of cells or percentage (with statistics) is provided.

Methodology

Sample preparation	Cells were washed with PBS and extracellularly stained with antibodies or mouse serum in PBS+10%FBS for 30 min at RT. For intracellular staining, cells were fixed and permeabilised with Fix&Perm (ThermoFisher Scientific) and incubated with the secondary fluorescent-conjugated antibody for 15 min at RT. For acquisition, cells were resuspended with PBS. All centrifugation steps were performed for 5 min at 400 x g.
Instrument	FACSCelesta Flow Cytometer
Software	FlowJo_v10.6.1 (BD)
Cell population abundance	Data provided in Supplementary Figure 1.
Gating strategy	The initial gating strategy used for Figures 1B and 2A involved a standard exclusion of debris by FSC-A/SSC-A and a selection of singlets by SSC-A/-SSC-H.

☐ Tick this box to confirm that a figure exemplifying the gating strategy is provided in the Supplementary Information.

Magnetic resonance imaging

Experimental design

Design type	n/a
Design specifications	n/a
Behavioral performance measures	n/a

Acquisition

Imaging type(s)	n/a
Field strength	n/a
Sequence & imaging parameters	n/a
Area of acquisition	n/a

Diffusion MRI ☐ Used ☐ Not used

Preprocessing

Preprocessing software	n/a
Normalization	n/a
Normalization template	n/a
Noise and artifact removal	n/a
Volume censoring	n/a

Statistical modeling & inference

Model type and settings	n/a
Effect(s) tested	n/a

Specify type of analysis: ☐ Whole brain ☐ ROI-based ☐ Both

Statistic type for inference (See Eklund et al. 2016)	n/a
Correction	n/a

Models & analysis

n/a	Involved in the study
☒	☐ Functional and/or effective connectivity
☒	☐ Graph analysis
☒	☐ Multivariate modeling or predictive analysis
